# Genomics of rapid ecological divergence and parallel adaptation in four tidal marsh sparrows

**DOI:** 10.1002/evl3.126

**Published:** 2019-07-16

**Authors:** Jennifer Walsh, Phred M. Benham, Petra E. Deane‐Coe, Peter Arcese, Bronwyn G. Butcher, Yvonne L. Chan, Zachary A. Cheviron, Chris S. Elphick, Adrienne I. Kovach, Brian J. Olsen, W. Gregory Shriver, Virginia L. Winder, Irby J. Lovette

**Affiliations:** ^1^ Fuller Evolutionary Biology Program Cornell Laboratory of Ornithology Ithaca New York 14850; ^2^ Department of Ecology and Evolutionary Biology Cornell University Ithaca New York 14853; ^3^ Division of Biological Sciences University of Montana Missoula Montana 59812; ^4^ Department of Forest and Conservation Sciences University of British Columbia Vancouver British Columbia T6T1Z4 Canada; ^5^ ’lolani School Honolulu Hawaii 96826; ^6^ Ecology and Evolutionary Biology University of Connecticut Storrs Connecticut 06269; ^7^ Department of Natural Resources and the Environment University of New Hampshire Durham New Hampshire 03824; ^8^ School of Biology and Ecology University of Maine Orono Maine 04469; ^9^ Department of Entomology and Wildlife Ecology University of Delaware Newark Delaware 19716; ^10^ Department of Biology Benedictine College Atchison Kansas 66002

**Keywords:** Adaptation, ecological speciation, new world sparrows, salt marsh

## Abstract

Theory suggests that different taxa having colonized a similar, challenging environment will show parallel or lineage‐specific adaptations to shared selection pressures, but empirical examples of parallel evolution in independent taxa are exceedingly rare. We employed comparative genomics to identify parallel and lineage‐specific responses to selection within and among four species of North American sparrows that represent four independent, post‐Pleistocene colonization events by an ancestral, upland subspecies and a derived salt marsh specialist. We identified multiple cases of parallel adaptation in these independent comparisons following salt marsh colonization, including selection of 12 candidate genes linked to osmoregulation. In addition to detecting shared genetic targets of selection across multiple comparisons, we found many novel, species‐specific signatures of selection, including evidence of selection of loci associated with both physiological and behavioral mechanisms of osmoregulation. Demographic reconstructions of all four species highlighted their recent divergence and small effective population sizes, as expected given their rapid radiation into saline environments. Our results highlight the interplay of both shared and lineage‐specific selection pressures in the colonization of a biotically and abiotically challenging habitat and confirm theoretical expectations that steep environmental clines can drive repeated and rapid evolutionary diversification in birds.

**Impact Summary**

Organisms that inhabit salt marshes are faced with extreme challenges and must cope with harsh, open environments as well as daily influxes of salt water into the system. Our whole‐genome comparisons among four species of sparrows demonstrate the important interplay between species‐specific and parallel signatures of selection in driving adaptation across an ecological gradient. Several lineage‐specific adaptations to salt marshes, particularly related to salt tolerance, suggest that selection of independent pathways may be important for allowing increased osmoregulatory function in salt marsh environments. Our results highlight the utility of a comparative genomics approach in characterizing the genomic basis of local adaptation and may aid in informing conservation strategies for threatened salt marsh endemics through the characterization of evolutionary potential.

Steep ecological gradients between saline and freshwater environments represent a potential barrier to the radiation of terrestrial species given the extreme physiological challenges present in saline environments (Greenberg and Maldonado 2006; Bayard and Elphick 2011; Le Moan et al. 2016; Ravinet et al. 2016). Adaptive variation in avian taxa that span such salinity gradients is well documented for both physiological and morphological traits (Grinnell 1913; Luttrell et al. 2015), including convergence in (1) bill size, as salt marsh birds have larger bills to facilitate greater heat exchange in harsh, open environments such as salt marshes (Grenier and Greenberg 2006; Greenberg and Olsen 2010; Tattersall et al. 2017); (2) modified kidney structure, as well as modified drinking behaviors in response to salt water, as salt marsh birds curb the volume of their drinking at saline concentrations below their osmotic tolerance, whereas their upland relatives do not (Poulson 1969; Goldstein 2006); and (3) coloration, as salt marsh birds are typically more melanic than their upland relatives, an adaptation potentially linked to UV protection and resistance to bacterial degradation (Greenberg and Droege 1990; Luttrell et al. 2015). Despite these known parallels in heritable phenotypic traits, neutral genetic differentiation between upland and salt marsh populations is notably low in many species, suggesting a strong role for ecological selection in salt marsh populations (Chan and Arcese 2002; Greenberg et al. 2016).

Salt marsh habitats have expanded in North America relatively recently, existing in their present locations for no more than a few thousand years, suggesting that colonization and adaptation to these habitats is itself recent (Malamud‐Roam et al. 2006). Therefore, salt marshes are an ideal ecosystem in which to investigate patterns of rapid evolution while providing unusually tractable settings for documenting ecological adaptation and speciation. We predict that intense, recent selection on loci of adaptive importance in an otherwise largely homogenous genomic background will make these replicated comparisons particularly powerful for identifying the underlying genomic basis of convergently adaptive traits. In addition, variation in levels of contemporary gene flow, as well as differences in colonization history between the replicated comparisons provides the opportunity to evaluate the role of demographic history in shaping the genomic landscape. By studying divergence across these steep ecological gradients across multiple lineages, we can explore the genetic architecture of convergent evolution and begin to answer the question: do taxa experiencing shared selective pressures adapt via *parallel selection on the same genes* and pathways or via *lineage‐specific selection on different genes* and pathways that ultimately lead to a similar functional endpoint?

Here, we use independent salt marsh colonizations by four Passerellidae sparrow lineages to disentangle the selective and demographic forces that have shaped their subsequent adaptation to physiologically challenging, saline environments. We specifically focus on the role of parallel (shared across lineages) versus lineage‐specific selection as drivers of salt marsh adaptations across all of these evolutionarily independent sparrow populations. Our comparative genomics approach is based on 80 whole genomes from four independent salt marsh specialists, each paired with their closest upland subspecies (Fig. 1 and Table S1): savannah sparrows (*Passerculus sandwichensis nevadensis* and *Passerculus sandwichensis beldingi*), Nelson's sparrows (*Ammospiza nelsoni nelsoni* and *Ammospiza nelsoni subvirgatus*), song sparrows (*Melospiza melodia gouldii* and *Melospiza melodia pusillula*), and swamp sparrows (*Melospiza georgiana georgiana* and *Melospiza georgiana nigrescens*). For each species comparison, upland populations inhabit freshwater marshes or meadows. The four salt marsh populations inhabit marshes that vary along the salinity gradient (i.e., true salt marsh habitats for song and savannah sparrows compared to more brackish marshes for Nelson's and swamp sparrows), however all of these populations are exposed to saline conditions. By studying replicate comparisons across these independent pairs of phylogenetically related taxa exposed to similar adaptive pressures, we substantially increase our statistical power for inferring the genetic basis of convergent adaptive variation and for testing questions about the scope and pattern of parallel evolution (i.e., adaptive traits arising through similar mechanisms; Schluter 1996; Wood et al. 2005; Manceau et al. 2010; Elmer and Meyer 2011).

**Figure 1:**
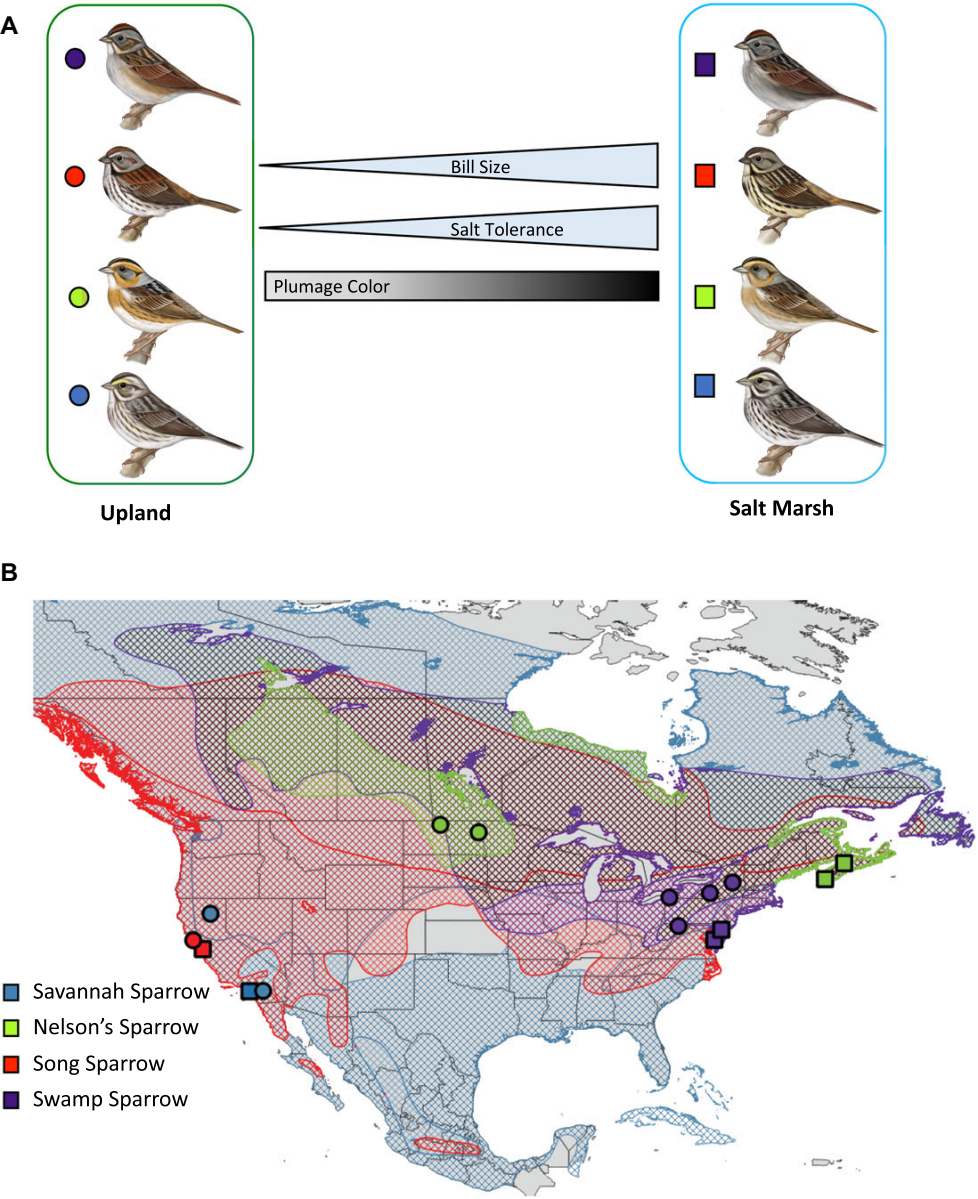
Sampling design and comparative framework for saltmarsh and upland sparrow groups. (A) Conceptual framework depicting the direction of phenotypic divergence between saltmarsh birds and their closest upland relatives (illustrations by Jillian Ditner 2019). (B) Range map depicts distribution of the four species: savannah sparrows (blue), Nelson's sparrows (green), song sparrows (red), and swamp sparrows (purple). Sampling locations for salt marsh (squares) and upland (circles) populations are included and correspond to the species colors described above.

## Methods

### SAMPLE COLLECTION

For genome resequencing, we sampled 80 individuals from inland and salt marsh populations of savannah, swamp, song, and Nelson's sparrows (Fig. 1 and Table S1). These comparisons included whole genome sequences from *M. m. gouldii* (*n* = 10), a subspecies of song sparrow that breeds in freshwater marshes along the California coast (Grinnell and Miller 1944), versus *M. m. pusillula* (*n* = 10), a subspecies endemic to salt marsh habitats in the San Francisco Bay; *Ammospiza nelsoni nelsoni* (*n* = 8), a subspecies of the Nelson's sparrow complex that inhabits freshwater marshes and meadows in the continental interior from eastern British Columbia to northern South Dakota, versus *A. n. subvirgatus* (*n* = 9), a subspecies that inhabits brackish and salt water marshes along the Atlantic coast from the Canadian Maritimes to southern Maine (Greenlaw and Rising 1994); *P. s. nevadensis* (*n* = 10), the subspecies of the savannah sparrow complex that breeds in grassy meadows and pastures in the Great Basin (Wheelwright and Rising 1993), versus *P. s. beldingi* (*n* = 10), a subspecies restricted to the Pacific coast salt marshes from Santa Barbara, CA to Rosario, Baja California, Mexico (Van Rossem 1947; Rising 2001); and *M. g. georgiana* (*n* = 12), the southern swamp sparrow that breeds in freshwater wetlands within formerly glaciated areas of eastern North America, versus *M. g. nigrescens* (*n* = 11), which breeds in brackish salt marshes of the Mid‐Atlantic United Sates (Greenberg and Droege 1990; Beadell et al. 2003).

### REFERENCE GENOME: ANNOTATION AND CHROMOSOME MAPPING

We used an existing reference genome from a male swamp sparrow (Deane‐Coe et al. 2018). To obtain more accurate information on chromosome position, scaffolds were assigned to chromosomes using the Chromosemble command in Satsuma (Grabherr et al. 2010) based on assigned chromosomes in the zebra finch (*Taeniopygia guttata*) reference genome. We annotated the swamp sparrow assembly with the MAKER pipeline version 2.31.8 (Cantarel et al. 2007). Gene models were created using the zebra finch. Ensemble protein and cDNA databases (downloaded July 7, 2017 from fhttp://useast.ensembl.org/Taeniopygia_guttata/Info/Index?redirect=no). Genes were predicted using SNAP using an iterative process to train the program. This produced a total of 13,733 gene models, which represented 71% of the genes annotated in the zebra finch genome.

### WHOLE‐GENOME RESEQUENCING AND VARIANT DISCOVERY

Genomic DNA was extracted using the DNeasy blood and tissue kit (Qiagen, CA, USA) and quantified with the Qubit dsDNA BR Assay Kit (Life Technologies). For the 57 song, savannah, and Nelson's sparrows, we prepared individually barcoded libraries using 1 µg of DNA following the TruSeq DNA PCR‐free library preparation kit protocol (San Diego, CA, USA), with an insert size of 350 bp. We pooled 24 libraries using concentrations of adapter‐ligated DNA determined through digital PCR into one group. The pooled libraries were sequenced on three Illumina NextSeq500 lanes at the Cornell Institute for Biotechnology core facility. The quality of individual libraries was assessed using FastQC version 0.11.5 (http://www.bioinformatics.babraham.ac.uk/projects/fastqc). Resequencing data from the 23 swamp sparrows have been previously published and were obtained from Deane‐Coe et al. (2018).

We performed sequence trimming, adapter removal, and quality filtering with AdapterRemoval version 2.1.1 (Lindgreen 2012). We allowed a minimum Phred quality score of 10 and merged overlapping paired‐end reads. We aligned all individuals to the swamp sparrow reference genome using the local option implemented in Bowtie2 version 2.2.8 (Langmead and Salzberg 2012). Alignment statistics were obtained using qualimap version 2.1.1 (Okonechnikov et al. 2015). Mean alignment rates for the four species comparisons were 87% (savannah sparrows), 85% (Nelson's sparrows), 83% (song sparrows), and 89% (swamp sparrows; Fig. S13). Mean coverage across all individuals was 4.2X (1.6X – 11X).

Resulting SAM files were converted to BAM files and sorted and indexed using Samtools version 1.3 (Li et al. 2009). We realigned around indels and fixed mate‐pairs using GATK version 3.5 (McKenna et al. 2010). SNP variant discovery and genotyping for the 80 resequenced individuals was performed using the unified genotyper module in GATK. We used the following filtering parameters to remove variants: QD < 2, FS > 40.0, MQ < 20.0, and HaplotypeScore > 12.0. Variants that were not biallelic had minor allele frequencies less than 5%, mean coverage less than 2X or more than 50X, and more than 20% missing data across all individuals were additionally filtered from the data set. To maximize the number of variants within species, we called variants for each comparison separately. This resulted in 13,025,553 SNPs for savannah sparrows, 2,680,218 SNPs for Nelson's sparrows, 2,333,568 SNPs for song sparrows, and 12,352,927 SNPs for swamp sparrows. We additionally used the same filtering parameters above to call SNPs in all individuals collectively. This resulted in 21,107,870 SNPs across all four sparrow species.

### DEMOGRAPHIC ANALYSES

To estimate the demographic history associated with each upland‐salt marsh split, we fit a range of demographic models to the joint SFS of the upland and salt marsh population for each of the four species in ∂a∂i version 1.7 (Gutenkunst et al. 2009). Each demographic model consisted of a split between the salt marsh and upland population and we modeled a series of demographic scenarios both with continuous and without gene flow between the two populations (see Fig. S14 for all models and parameters). To generate the joint‐SFS input for ∂a∂I, we filtered VCF files output from GATK for each species using the program VCFtools (Danecek et al. 2011) to include SNPs with mean coverage greater than 8X and less than 50X that were present across all individuals and were biallelic. We did not filter based on minor allele frequency as these rare variants are important for demographic inference. Next, we removed SNPs that mapped to the z‐chromosome, as the z‐chromosome has a smaller effective population size than the autosomes. Finally, we excluded all SNPs that were mapped to exons or intervals within 100 kb of exons to produce a final, putatively neutral data set to reduce bias due to selection or linked selection, which can significantly bias demographic inference (Schrider et al. 2016). This filtering resulted in data sets of 59,586, 128,803, 25,267, and 25,597 SNPs for savannah, swamp, Nelson's, and song sparrows, respectively. ∂a∂i input files were created from VCF files using perl script developed by Kun Wang (https://github.com/wk8910/bio_tools/blob/master/01.dadi/00.convertWithFSC/convert_vcf_to_dadi_input.pl). The swamp sparrow genome was used as an outgroup to polarize the joint‐SFS for savannah, Nelson's, and song sparrows. For models including the two populations of swamp sparrows, we ran demographic models using the folded SFS due to the absence of a genome from an appropriate outgroup for this pair.

For each species and all models, 10 optimizations were run from different starting parameters using the perturb function in ∂a∂i with max number of iterations set to 10 and the best fit model identified based on the highest log‐likelihood value. We calculated demographic parameter values from the estimated value of theta (4NeµL; L is sequence length) based on a 1‐year generation time for all sparrow species (Mowbray 1997; Arcese et al. 2002; Wheelwright and Rising 2008; Shriver et al. 2011) and the average mutation rate for Passeriformes: 3.3 × 10^−9^ substitutions/site/year (Zhang et al. 2014). We calculated uncertainty for parameter estimates using a nonparametric bootstrapping approach: sampling with replacement SNPs from different chromosomes, generating frequency spectra from 100 resampled SNP data sets, and using these spectra to calculate parameter uncertainties using the Godambe information matrix (GIM) in ∂a∂i (Gutenkunst et al. 2009). We used an Akaike information theoretic approach to rank the demographic models. Given the likely presence of linked‐SNPs within the data set, optimization in ∂a∂i is based on composite likelihoods and AIC analysis of composite likelihoods can lead to erroneous support for more complex models. To account for this bias, we performed a likelihood ratio test with an adjustment based on the GIM to identify the best fit model to each data set (Coffman et al. 2015).

### DEMOGRAPHIC SIMULATIONS

We simulated a SNP data set based on the best‐fit demographic model and parameter estimates from ∂a∂i within the coalescent simulator msprime (Kelleher et al. 2016) to account for the potential influence of the inferred demographic history on the genomic distribution of summary statistics used to infer outliers (i.e., *F*_ST_, pi, and Tajima's *D*). For each species, we simulated 1072, 1 mb regions to approximate the length of the whole genome sequences. For all simulations, we assumed a passerine mutation rate of 3.3 × 10^−9^ (Zhang et al. 2014) and a recombination rate of 0.14 cm/mb (1.4 × 10^−8^ in msprime units) estimated from Estrildidae finches (Singhal et al. 2015). We calculated *F*_ST_ for each SNP, and nucleotide diversity (pi) and Tajima's *D* for 25 kb windows across the simulated data set within the python package scikit‐allel (Alistair and Harding 2017). We then inferred the mean, SD, and 95th and 99th percentiles for the distribution of these summary statistics to compare with observed data from each species pair. We compared the cutoff used to identify outliers with the simulated distribution for each summary statistic to ensure that cutoffs were conservative and neutral demography did not bias our inferences of outlier loci. Second, we ran a series of simulations to account for the high uncertainty associated with some parameters. For each species pair, we simulated a SNP data set and calculated the *F*_ST_ distribution as above 1000 times while randomly drawing parameter values for the demographic model from a uniform distribution bounded by the upper and lower 95% CIs of each parameter. We inferred the 95th and 99th percentile for each simulated *F*_ST_ distribution and then compared the 99th percentile of the distribution of these percentiles from all 1000 simulations to the empirical cutoff (mean *F*_ST_ + 5 × SD).

### POPULATION GENOMICS AND PATTERNS OF DIVERGENCE

For each of the four species’ upland‐saltmarsh comparisons, PCA was performed on all SNPs using the snprelate package in R (Team 2016). We calculated nucleotide diversity (pi), individual heterozygosity, and Tajima's *D* using 25 kb windows in VCFtools. We characterized genome‐wide patterns of divergence by calculating *F*_ST_ values using VCFtools. We calculated *F*_ST_ for both 25 kb windows and for individual SNPs. Divergent peaks were visualized using Manhattan plots, which were constructed using the R package qqman. Within each species comparison, we classified windows as divergent if mean *F*_ST_ was more than 5 standard deviations above the genome‐wide mean. We report results based on a 5 standard deviation cutoff after simulations based on demographic data confirm that this is a conservative approach to identifying outliers. To identify potential selective sweeps in elevated regions of differentiation, we compared values of Tajima's *D* and pi inside and outside of outlier windows (i.e., assessing whether regions of elevated differentiation had corresponding dips in Tajima's *D* and pi). To do this in a way that accounted for differences in sample size between neutral and elevated windows, we used a permutation test to compare the Tajima's D and pi estimates from the outlier windows to the same number of randomly chosen neutral windows. Finally, differentiated peaks were inspected in Geneious version 9.1.5 (Kearse et al. 2012) and compiled a list of gene models within 50 kb of each region and obtained information on these annotations from the UniProt database (http://www.uniprot.org/). To characterize putative candidate genes, we used ontology information from the Zebra Finch Ensemble database. We also compared genes to several previously published candidate gene lists (Islam et al. 2013; Ferchaud et al. 2014).

### ENRICHMENT ANALYSIS

We performed GO analyses of candidate genes (mean *F*_ST_ greater than 5 standard deviations above the genome‐wide mean) for each species pair using the Web‐based GOfinch tool (http://bioinformatics.iah.ac.uk/tools/Gofinch). We provided gene‐level annotations from Ensembl and used Fisher and Hypergeometric tests of enrichment for terms in our input list.

## Results

### GENOME‐WIDE PATTERNS OF DIVERGENCE BETWEEN UPLAND AND SALT MARSH ENVIRONMENTS

**Figure 2:**
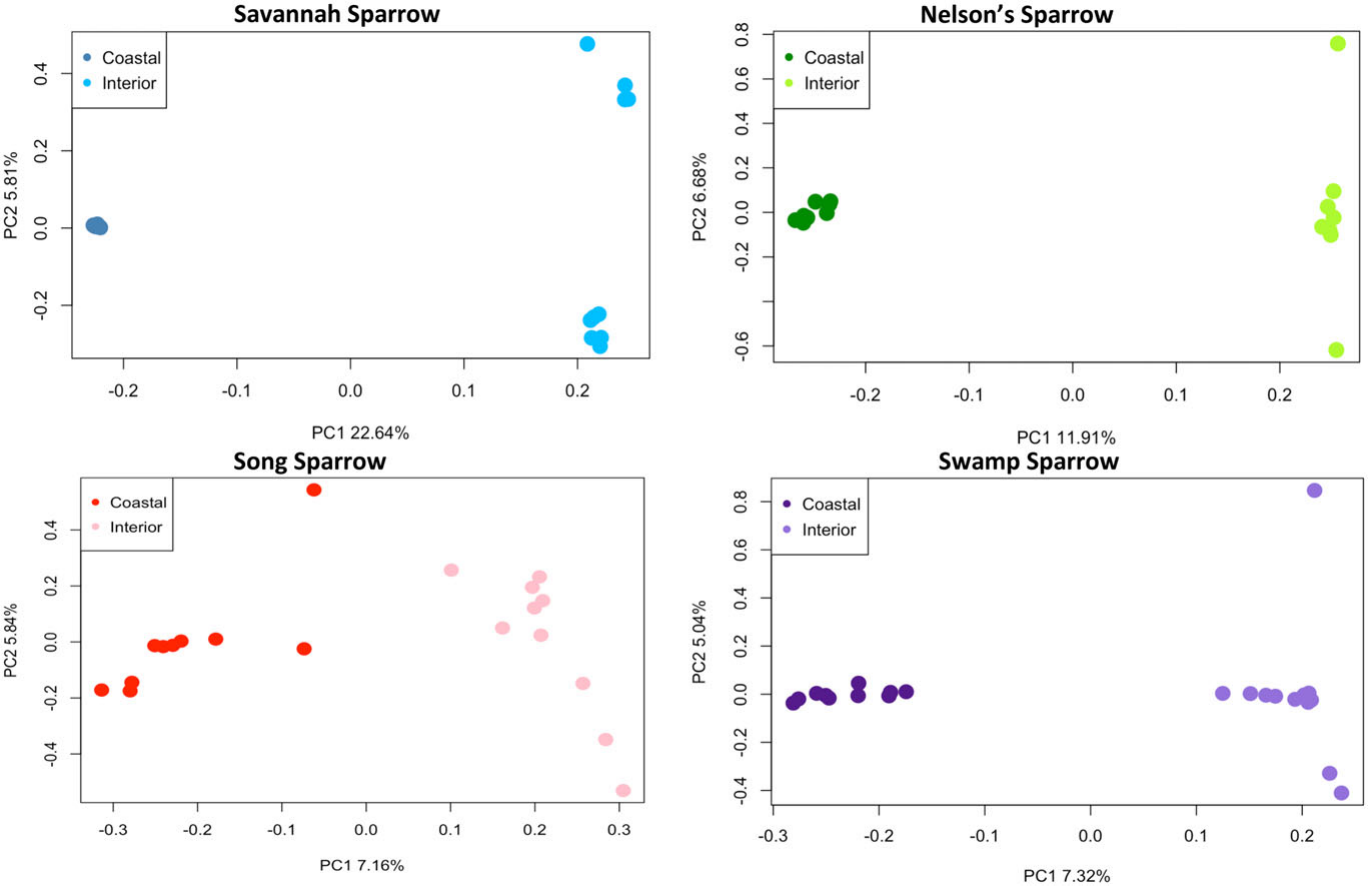
Genome‐wide patterns of divergence between salt marsh and upland populations of four sparrow species pairs. PCA plots show clear splits between salt marsh (dark) and upland (light) populations of each species: savannah sparrows (*n* = 20; blue), Nelson's sparrows (*n* = 17; green), song sparrows (*n* = 20; red), and swamp sparrows (*n* = 23; purple).

For each of the four species pairs, we documented a clear division between salt marsh and upland populations based on 2.3–13 million single‐nucleotide polymorphisms (SNPs; Fig. 2 and Fig. S2). Salt marsh and upland populations consistently split along the first PC axis (% variation explained: 23% for savannah sparrows, 12% for Nelson's sparrows, 7.2% for song sparrows, and 7.3% for swamp sparrows). Genetic differentiation between salt marsh and upland populations was further supported by genome‐wide estimates of *F*_ST_: 0.02 (song sparrows), 0.03 (swamp sparrows), 0.07 (Nelson's sparrows), and 0.26 (savannah sparrows; Figs. S3–S7). Although the magnitude of differentiation between upland and salt marsh populations varied (as seen in the range of *F*_ST_ estimates), we found a strong delineation between all pairs of salt marsh and upland populations, highlighting the clear potential for similar processes of ecological divergence across the freshwater‐saline habitat barrier. Estimates of pi, heterozygosity, and Tajima's *D* were notably similar between salt marsh and upland populations (Table S2).

### CHARACTERIZATION OF DEMOGRAPHIC HISTORY ASSOCIATED WITH SALT MARSH COLONIZATION

**Figure 3:**
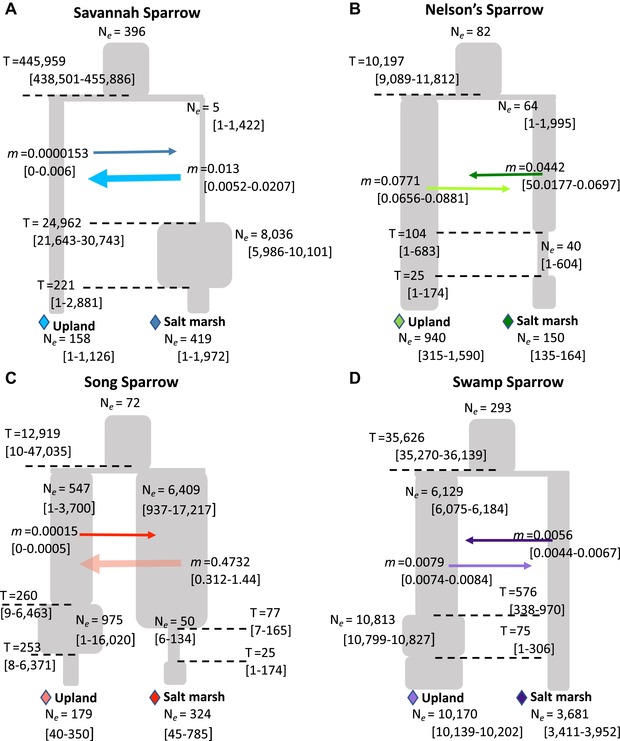
Demographic history of salt marsh and upland populations of four sparrow species pairs. Each panel presents estimates from *dadi* for each of the four species. Estimates include effective population sizes (*N*_e_) and divergence times (dotted lines; T). Gene flow (m) is shown as arrows between lineages (migration is continuous and bidirectional). Migration rates refer to the proportion of immigrants within the population the arrow points to. All parameter estimates are expressed as means with the 95% confidence intervals in brackets. Interpretation should be focused on relative comparisons between parameter estimates, rather than actual value of the estimates.

Demographic processes, including divergence time, contemporary gene flow, and fluctuations in population size, can shape genomic landscapes in conjunction with selective pressures. We inferred demographic history from the joint site frequency spectrum (SFS) of nongenic SNPs (i.e., SNPs > 100 kb from any exon) to account for the influence of species‐specific, neutral demography on patterns of genomic divergence. Log‐likelihoods for the top demographic models ranged from –1376.21 in song sparrows to –4812.24 in savannah sparrows with residual error normally distributed largely between –30 to 30. In many species, there were more SNPs distributed along the center of the joint‐SFS than inferred by the top model, suggesting the model may be underestimating migration rate (Fig. S1). The best‐fit models from demographic analyses indicate that all salt marsh populations (except for swamp sparrow) experienced fluctuations in effective population size following divergence from upland relatives. Population bottlenecks appear to have occurred recently in most species. This may be linked to anthropogenic development along both coasts (Bertness et al. 2002) where up to 90% of the available tidal marsh habitat has been lost in the past 150 years (Takekawa et al. 2006). Divergence times varied significantly among species, with divergence between salt marsh and upland Nelson's and song sparrow populations occurring most recently (∼10,000 and ∼13,000 ya, respectively) and Savannah sparrow populations diverging the earliest ∼445,000 ya. These divergence times vary among species in similar ways to principle components analysis (PCA) and *F*_ST_ results reported above. Finally, a model of continuous gene flow between upland and salt marsh populations was supported for all species (Fig. 3; Fig. S1, Supporting Information Notes, and Table S3). Support for continuous gene flow model could reflect an actual history of continuous gene flow or pulses of intermittent gene flow, but we did not attempt to distinguish between these scenarios in our model.

### DETECTING REGIONS OF DIVERGENCE BETWEEN SALT MARSH AND UPLAND PAIRS

**Figure 4:**
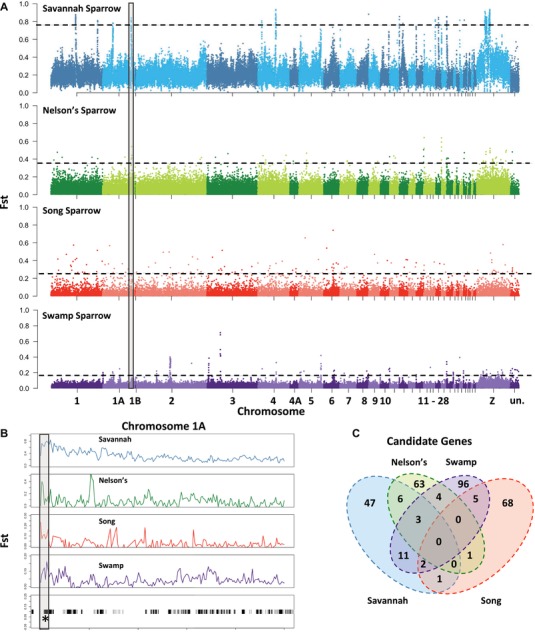
Genome‐wide divergence between upland‐salt marsh pairs of sparrows. (A) Manhattan plots depict the genomic landscape (*F*_ST_) in nonoverlapping 25 kb windows for each comparison. Dashed line represents 5 standard deviations above the genome‐wide mean. (B) Example of parallel evolution where a peak is shared in three of the four comparisons (savannah, Nelson's, and swamp). Shared peak is boxed in gray in panels (A) and (B). Bottom graph shows the genes housed in this region (genes are denoted by gray and black boxes); * denotes the candidate gene—SLC41A2—under selection. (C) Venn diagram depicting the number of unique and shared candidate genes across the four species comparisons.

We compared *F*_ST_ values to identify divergent regions between the genomes of dyads of upland‐salt marsh sparrows (Fig. 4A), considering 25 kb windows with a mean *F*_ST_ (averaged over all SNPs contained in window) greater than 5 standard deviations above the genome‐wide mean to be elevated (Fig. 4A). To ensure that this *F*_ST_ cutoff would be robust to the inferred demographic history above, we simulated SNPs from a similarly sized whole genome data set based on the best‐fit demographic model and parameter estimates. We then estimated the distribution of *F*_ST_ values for the simulated SNP data set and calculated the *F*_ST_‐cutoff as 5 standard deviations above the simulated mean *F*_ST_ (Fig. S8). Comparison of the simulated and empirical cutoffs revealed substantial overlap between the total number of outliers identified. Across all four of our species comparisons, 75% of the candidate windows identified using the 5 standard deviations cutoff in the empirical data set exhibited *F*_ST_ estimates that either matched or were greater than the simulated estimates. We note that with the cutoff approach used to detect outliers in our empirical data set, some candidate genes may have been missed (namely in savannah and Nelson's sparrows), whereas some candidate regions should be interpreted more cautiously. However, several of these regions of elevated divergence exhibit corresponding dips in Tajima's *D* and pi (see below) and thus offer candidate genes that warrant further investigation. We also accounted for uncertainty in parameter estimates by performing an additional 1000 simulations randomly sampling from a uniform distribution bounded by the 95% confidence intervals around each parameter estimate. For all species, the 95th percentile for each of the 1000 simulated *F*_ST_ distributions fell below the empirical *F*_ST_ cutoff (Fig. S9); however, for some species (e.g., swamp and song sparrow) the empirical cutoff did not exceed the 99th percentile under some simulations (Fig. S10). Together these simulations suggest that *F*_ST_ outlier windows cannot be explained by neutral demographic processes and thus likely reflect regions under selection. Because our 5 standard deviation empirical cutoff was determined to be a conservative approach, overall (Fig. S8) and generally robust to parameter uncertainty, we report results based on this method.

### SIGNALS OF PARALLEL VERSUS LINEAGE SPECIFIC EVOLUTION

Using the above method, we identified elevated windows of differentiation between salt marsh and upland pairs of each species. Elevated window frequency varied somewhat among the four comparisons: 78 elevated windows between populations of Nelson's sparrows, 112 between populations of song sparrows, 155 between populations of swamp sparrows, and 223 between populations of savannah sparrows. In all four cases, these differentiated windows in aggregate are a very small fraction of the entire genome (<0.005% across all comparisons). Several, but not all, of these differentiated windows harbored candidate genes with associated annotations: 43 elevated windows with annotated genes (55%) in Nelson's sparrows, 46 elevated windows (41%) in song sparrows, 67 elevated windows (43%) in swamp sparrows, and 50 elevated windows (22%) in savannah sparrows.

We classified regions as shared if multiple paired comparisons identified the same gene within 50 kb of an elevated window. We consider these *shared regions* of elevated differentiation between *multiple* upland‐saltmarsh pairs as signatures of *parallel selection*. In contrast, we consider any *independent regions* of elevated differentiation within *single* upland‐saltmarsh pairs as signatures of *lineage‐specific selection*. Furthermore, we consider gene regions that exhibit both elevated differentiation and corresponding dips in Tajima's *D* as our most compelling candidates for both parallel and lineage‐specific selection in this system (Tajima 1989; Beaumont 2005). Several elevated regions exhibited both elevated differentiation and dips in Tajima's *D* (here, defined as negative Tajima's *D* estimates): 14 (32%) of the regions identified in Nelson's sparrows, 15 (32%) of regions in song sparrows, 23 (46%) of the regions in savannah sparrows, and 23 (34%) of the regions identified in swamp sparrows. More broadly, across all species comparisons combined, we saw a significant reduction in Tajima's *D* in differentiated windows (mean = 0.75) compared to a random subset of neutral windows (mean = 1.08; permutation test, *F*‐statistic = 26.94, *P* < 0.001; Fig. S11). Similarly, across all of our comparisons combined, we saw a significant reduction in nucleotide diversity in differentiated windows (mean = 0.0005) compared to a random subset of neutral windows (mean = 0.002; permutation test, *F*‐statistic = 280.7, *P* < 0.001; Fig. S12). Significant reduction in these estimates in conjunction with elevated estimates of *F*_ST_ offer additional support for selection (both parallel and lineage specific) between salt marsh and upland comparisons.

Under our definition of parallel selection, we found no regions of elevated differentiation between salt marsh and upland populations that were shared among all four comparisons. However, we did identify several elevated windows between upland and saltmarsh populations that were shared among two or three such pairs (Fig. 4C and Table S4). If we consider the regions of shared differentiation that have associated gene annotations, we identified a total of 33 candidate regions for parallel selection (Table S4). Of these 33 candidate genes, we identified 16 genes (48%) as having a putative role in tidal marsh adaptation based on literature reviews and *a priori* knowledge (Table S4). Several regions under putative parallel selection offer compelling associations with salt marsh adaptations known *a priori*, including genes linked to heat tolerance, bill size, melanogenesis pathways, and osmotic regulation (Table S4).

**Figure 5:**
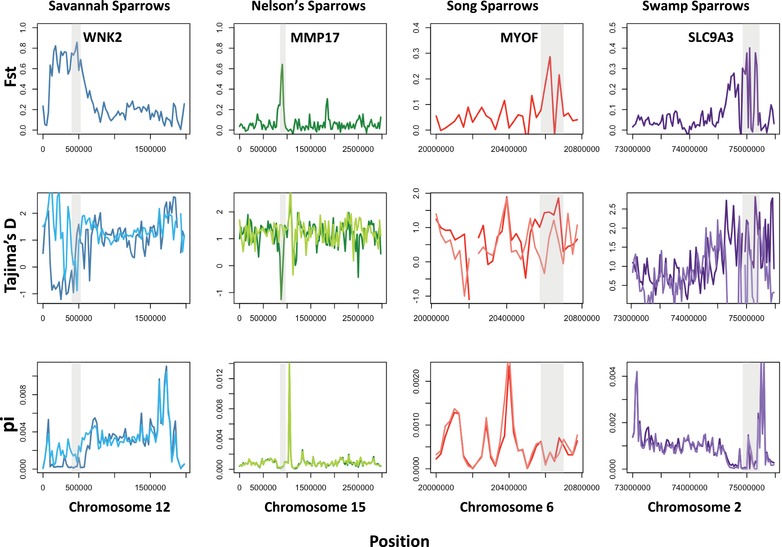
Regions of elevated divergence unique to each of four sparrows suggest independent adaptation to salt marsh environments. Top row: *F*_ST_ for putative regions under selection in savannah, Nelson's, song, and swamp sparrows. Candidate gene associated with each peak is labeled in each plot. Middle and bottom rows: Tajima's *D* and nucleotide diversity for the same elevated region, presented for coastal (darker colors) and interior (lighter colors) populations for each species. In all plots, elevated windows are shaded in gray. The four genes presented in this figure (WNK2, MMP17, MYOF, and SLC9A3) are lineage‐specific candidates for osmoregulatory function, demonstrating a mechanism for unique genetic pathways resulting in phenotypic convergence.

Although we found some gene candidates that were shared across our multispecies comparisons, many regions exhibited elevated differentiation within only a single dyad of populations, suggesting an important role for novel genetic solutions that result in a convergent salt marsh phenotype (Fig. 5, Tables S5–S8). We performed GO analyses of candidate genes for each species pair using the Web‐based GOfinch tool (http://bioinformatics.iah.ac.uk/tools/Gofinch) and in each species comparison, we observed significant enrichment for genes that participate in pathways important for osmotic regulation, but the specific pathways differ among them. Enriched pathways include the positive regulation of sodium ion transport in Nelson's sparrows (*P* < 0.001), regulation of Rho GTPase activity in song sparrows (*P* = 0.0029), regulation of actin cytoskeleton organization in savannah sparrows (*P* = 0.0024), and regulation of JNK cascades in swamp sparrows (*P* = 0.0052). This role for novel genetic solutions for osmoregulatory function was further supported by candidate genes identified via examination of genomic regions exhibiting elevated divergence. We documented elevated differentiation for 15 gene regions linked to osmoregulation in savannah sparrows (30% of the total candidate regions identified; Table S5), 18 gene regions in Nelson's sparrows (42% of the total candidate regions identified; Table S6), 19 elevated gene regions in song sparrows (41% of the total candidate regions identified; Table S7), and 23 elevated gene regions in swamp sparrows (34% of the total candidate regions identified; Table S8). Consistent with *a priori* predictions, genes associated with these regions are linked to morphological and physiological features that are subject to selection for improved performance in salt marsh environments (Tables S5–S8).

## Discussion

### DEMOGRAPHIC PROCESSES INFLUENCE THE GENOMIC LANDSCAPE

Analysis of putatively neutral regions of the genome revealed meaningful variation in demographic history among species that could influence unique patterns of genomic divergence in each species. Divergence time between salt marsh and upland populations varied nearly 45‐fold between Nelson's (∼10,000 ya) and Savannah sparrows (∼446,000 ya). These differences in divergence time are reflected in the greater elevation of genomic divergence and identification of more differentiated windows in Savannah (122 windows) versus Nelson's sparrow (78 windows). However, greater overall genomic differentiation in Savannah sparrows was not associated with the identification of a greater number of candidate genes (e.g. Fig. 4C), which might be predicted if longer exposure to selective pressures allowed for the fixation of more adaptive mutations relative to younger salt marsh populations (McGee et al. 2016). Migration rates were largely symmetrical between upland and salt marsh populations of Nelson's and swamp sparrows, with only slightly greater gene flow from upland to salt marsh populations. In contrast, Savannah and song sparrows exhibited greater gene flow in the opposite direction from saline in to freshwater populations. Differences in gene flow patterns among species may also contribute to genomic landscape differences. For example, song and Nelson's sparrows exhibit similar divergence times and bottleneck histories, but the saltmarsh and upland Nelson's sparrows show more defined genomic peaks of divergence and overall higher baseline *F*_ST_ relative to the song sparrow comparison. It is possible that the higher levels of gene flow between song sparrow populations is resulting in the erosion of divergence peaks (Ravinet et al. 2017); however, a number of other factors (e.g., recombination rate and background selection) could also contribute to these differences and will require further exploration to establish causality (Duranton et al. 2018). Finally, all species except for swamp sparrows experienced bottlenecks in salt marsh populations. Bottlenecks can erode genetic diversity and elevate *F*_ST_ differentiation between populations (Pavlidis et al. 2012). Despite this potential bias, we do not identify greater numbers of candidate regions in sparrow populations experiencing a bottleneck relative to swamp sparrows (e.g., Fig. 4C). Moreover, simulations show that for all species that experienced a bottleneck the simulated *F*_ST_ distribution based on neutral demographic history showed a lower mean *F*_ST_ and cutoff (5 standard deviations greater than the mean) relative to the empirical *F*_ST_ distribution (Fig. S8). These patterns were also robust to uncertainty around parameter estimates for species experiencing bottlenecks in salt marshes (Fig. S9). Together these results suggest that patterns of genomic divergence cannot be accounted for by neutral demographic processes alone and that selection likely played a key role in shaping the observed genomic landscape.

### SIGNALS OF PARALLEL EVOLUTION

Several regions under putative parallel selection offer compelling associations with salt marsh adaptations (Table S4). One such example, gene SLC41A2, lies in a region exhibiting elevated differentiation in Nelson's, savannah, and swamp sparrows (Fig. 4B and Table S4). SLC41A2 functions as a membrane Na^+^/Mg^2+^ transporter. Mg^2+^ and Na^+^ are among the most abundant cations in salt water, with SLC41A2 identified as a Na^+^/Mg^2+^ exchanger that is highly expressed in the kidney of saltwater acclimated puffer fish compared to closely related freshwater species (Islam et al. 2013). We found similar patterns of parallel selection of 11 additional gene candidates that are functionally linked to osmotic regulation and response to salt stress (Table S4; DAB2, MAPK81P3, PPIP5K1, PPIP5K2, RBM12, TAF12, TMEM161B, ARHGAP5, DLC1, EPB41L4A, PAM). Most of these genes function in transmembrane and vesicle transport as well as cellular stress response to osmotic conditions. Many of these genes under putative parallel selection have been previously identified in comparative work on marine versus freshwater fish, including genes implicated in freshwater adaptation in sticklebacks (*Gasterosteus aculeatus*; TAF12; Ferchaud et al. 2014) and in salt water adaptation in the saltmarsh sparrow (*Ammospiza caudacutus;* Walsh et al. 2018), and likely represent important genomic mechanisms underlying osmoregulatory adaptation across freshwater‐saline gradients.

Another notable candidate was RBM39, which lies in a region exhibiting elevated differentiation in Nelson's, savannah, and swamp sparrows (Table S4). This gene functions in the negative autoregulation of BMP4 (Faherty et al. 2016); in birds, the expression and regulation of BMP is important in the diversification of bill shapes (Abzhanov et al. 2004; Wu et al. 2004; Helms and Brugmann 2007). In these three sparrows, BMP regulation may be a key genetic mechanism underpinning increased bill sizes in salt marsh sparrows, which in turn enables increased dry heat dissipation from the bill surface and reduced reliance on evaporative cooling in freshwater‐limited environments (Greenberg et al. 2012).

### NOVEL GENETIC SOLUTIONS WITHIN SPECIES LEAD TO A CONVERGENT SALT MARSH PHENOTYPE

Although we found some gene candidates that were shared across our multispecies comparisons, many regions exhibited elevated differentiation within only a single dyad of populations. For example, we identified enriched pathways that include the positive regulation of Rho GTPase activity, actin cytoskeleton organization, and of JNK cascades. Ion transporters are important components of osmoregulatory response and JNK signaling cascades have been shown to be activated by hyperosmotic stress (Koh et al. 2001). Enrichment of Rho GTPase activity in song sparrows and regulation of actin cytoskeleton organization in savannah sparrows suggests different pathways resulting in potentially similar functions. In response to hyperosmotic stress, cells can attempt to restore normal volume through regulatory volume increase/decrease; this is generally achieved through ion transport (Di Ciano‐Oliveira et al. 2006). Alternatively, cells can resist volume change by reinforcing cell structure via cytoskeletal reorganization (Di Ciano‐Oliveira et al. 2006); pathways linked to actin cytoskeleton organization and Rho GTPase activity are important putative pathways for this second response. Taken together, this suggests that these pathways are common targets of selection, but the specific genic targets of selection within the pathways differ among species.

In addition to the enriched pathways above, each species dyad exhibited multiple loci with elevated differentiation that have putative links to osmoregulation (Fig. 5). Of the gene regions exhibiting elevated divergence in savannah sparrows, we identified the candidate WNK2 (window averaged *F*_ST_ = 0.77), which acts as an activator and inhibitor of sodium‐coupled chloride cotransporters and appears to be an important component of an essential pathway for regulating cell volume in response to osmotic stress (Kahle et al. 2010). In Nelson's sparrows, we documented elevated differentiation for a region linked to the gene MMP17 (window averaged *F*_ST_ = 0.64), which is linked to both drinking behavior and kidney function and is expressed in the region of the brain responsible for regulating thirst in mice (Srichai et al. 2011). In song sparrows, we identified the candidate MYOF (window averaged *F*_ST_ = 0.28); differential expression of MYOF has been documented in response to being transferred between fresh and salt water in fish (*Scatophagus argus*; Su et al. 2016). Lastly, in swamp sparrows we observed elevated differentiation in a region associated with the gene SLC9A3 (window averaged *F*_ST_ = 0.37), which is involved in sodium ion import across the plasma membrane and was found to be differentially expressed in freshwater versus salt water sticklebacks (*G. aculeatus*; Gibbons et al. 2017). Considering these findings collectively, we identified a pervasive role for the putative selection of independent genes and/or pathways that allow for increased salt tolerance in sparrow populations that have colonized salt marshes, rather than shared or replicated selection for the same pathways across species.

### CONCLUSIONS AND BROADER IMPLICATIONS

In this naturally replicated set of New World sparrows that have experienced similar selection pressures during salt marsh adaptation, we have identified both parallel and novel targets of putative selection that may underlie solutions to osmotic stress, including candidate genes linked to the restoration of cell volume, resistance to cell volume changes, and behavioral avoidance of salt water. These findings contribute to a growing list of candidate genes linked to salt tolerance and osmoregulatory function (Kahle et al. 2010; Islam et al. 2013; Ferchaud et al. 2014; Gibbons et al. 2017), suggesting a polygenic nature of a complex set of physiological and behavioral phenotypes that lead to adaptation in salt water environments. Because we only identified a small subset of the adaptive gene candidates exhibiting parallel evolution in multiple lineages, we hypothesize that a lack of shared standing variation among the four focal species may have been a more important determinant of lineage‐specific (novel) versus parallel (shared) adaptation across the saline‐freshwater gradient than were these species’ shared ecological constraints (Yeaman et al. 2016). As such, additional studies on phylogenetically similar species may be useful in further understanding patterns of lineage‐specific versus parallel selection across environmental gradients.

Our results illustrate how a comparative approach can clarify the variation that selection and demography generate in a genomic landscape in the face of shared selective pressures. Demographic parameters, including divergence time, gene flow, and effective population sizes, can dramatically shift patterns across the genome (Elmer and Meyer 2011) and multispecies comparisons have allowed us to document ways in which combinations of these factors can shape our observations. Characterizing the genomic basis of adaptation across salt marsh‐upland habitats in conjunction with information on population changes over time will inform conservation strategies for threatened salt marsh endemics. Activities such as captive breeding and preservation of evolutionary potential will depend heavily on our understanding of locally adapted genotypes, particularly in vulnerable ecosystems such as tidal salt marshes where salt‐adapted populations may be irreplaceable over ecological time frames.

Associate Editor: Z. Gompert

## Supporting information


**Extended Data Figures S1**. Comparison of the observed joint‐SFS for each species (top left) to the simulated spectra of the best‐fit model and estimated parameter values (top right).
**Extended Data Figure S2**. PCA plot based on approximately 21 million SNPs for all four species (and eight subspecies) combined.
**Extended Data Figure S3**. Histograms of *F*_ST_ estimates for individual SNPs for each of the four species comparisons.
**Extended Data Figure S4**. Descriptive statistics for coastal and interior populations of savannah sparrows.
**Extended Data Figure S5**. Descriptive statistics for coastal and interior populations of Nelson's sparrows.
**Extended Data Figure S6**. Descriptive statistics for coastal and interior populations of song sparrows.
**Extended Data Figure S7**. Descriptive statistics for coastal and interior populations of swamp sparrows.
**Extended Data Figure S8**. Simulated *F*_ST_ distributions for each species based on neutral demographic history inferred with ∂a∂i.
**Extended Data Figure S9**. Distribution of 95th percentiles of the *F*_ST_ distribution from 1000 simulated SNP data sets.
**Extended Data Figure S10**. Distribution of 99th percentiles of the *F*_ST_ distribution from 1000 simulated SNP data sets.
**Extended Figure S11**. Distribution of Tajima's D estimates for each species comparison for elevated (red) and neutral (genome‐wide; blue) windows.
**Extended Figure S12**. Distribution of nucleotide diversity estimates for each species comparison for elevated (red) and neutral (genome‐wide; blue) windows.
**Extended Data Figure S13**. Boxplots depicting the percentage of reads mapped to the swamp sparrow reference genome (color coded by species).
**Extended Figure S14**. Different demographic models fit to joint site frequency spectrum of upland (light gray) and tidal marsh populations (dark gray).
**Extended Data Table S1**. Information and sampling locations for individual sparrows analyzed in this study.
**Extended Data Table S2**. Average observed heterozygosity, nucleotide diversity, and Tajima's *D* for coastal and interior populations of savannah, Nelson's, song, and swamp sparrows.
**Extended Data Table S3**. Parameter estimates for the best‐fit demographic model from ∂a∂i.
**Extended Data Table S4**. List of shared candidate genes in two or three species pairs identified through a comparative genomics approach.
**Extended Data Table S5**. Candidate genes identified through whole genome comparisons of freshwater and salt water populations of Savannah sparrows.
**Extended Data Table S6**. Candidate genes identified through whole genome comparisons of freshwater and salt water populations of Nelson's sparrows.
**Extended Data Table S7**. Candidate genes identified through whole genome comparisons of freshwater and salt water populations of Song sparrows.
**Extended Data Table S8**. Candidate genes identified through whole genome comparisons of freshwater and salt water populations of Swamp sparrows.
