# Supplementary material for: Genomics of rapid ecological divergence and parallel adaptation in four tidal marsh sparrows
Source: Evol Lett. 2019 Jul 16;3(4):324–38. doi: 10.1002/evl3.126 (PMC6675146; doi:10.1002/evl3.126)
Supplement: Supplementary file 1 — Extended Data Figures S1. Comparison of the observed joint‐SFS for each species (top left) to the simulated spectra of the best‐fit model and estimated parameter values (top right). Extended Data Figure S2. PCA plot based on approximately 21 million SNPs for all four species (and eight subspecies) combined. Extended Data Figure S3. Histograms of F ST estimates for individual SNPs for each of the four species comparisons. Extended Data Figure S4. Descriptive statistics for coastal and interior populations of savannah sparrows. Extended Data Figure S5. Descriptive statistics for coastal and interior populations of Nelson's sparrows. Extended Data Figure S6. Descriptive statistics for coastal and interior populations of song sparrows. Extended Data Figure S7. Descriptive statistics for coastal and interior populations of swamp sparrows. Extended Data Figure S8. Simulated F ST distributions for each species based on neutral demographic history inferred with ∂a∂i. Extended Data Figure S9. Distribution of 95th percentiles of the F ST distribution from 1000 simulated SNP data sets. Extended Data Figure S10. Distribution of 99th percentiles of the F ST distribution from 1000 simulated SNP data sets. Extended Figure S11. Distribution of Tajima's D estimates for each species comparison for elevated (red) and neutral (genome‐wide; blue) windows. Extended Figure S12. Distribution of nucleotide diversity estimates for each species comparison for elevated (red) and neutral (genome‐wide; blue) windows. Extended Data Figure S13. Boxplots depicting the percentage of reads mapped to the swamp sparrow reference genome (color coded by species). Extended Figure S14. Different demographic models fit to joint site frequency spectrum of upland (light gray) and tidal marsh populations (dark gray). Extended Data Table S1. Information and sampling locations for individual sparrows analyzed in this study. Extended Data Table S2. Average observed heterozygosity, nucleotide diversity, and Tajima [file EVL3-3-324-s001.docx]

**EXTENDED DATA:**

**Supplementary Figures:**

**Extended Data Figures 1:** Comparison of the observed joint-SFS for each species (top left) to the simulated spectra of the best-fit model and estimated parameter values (top right). Lower two panels for each species show residuals of fitting observed to simulated data and their distribution.

**
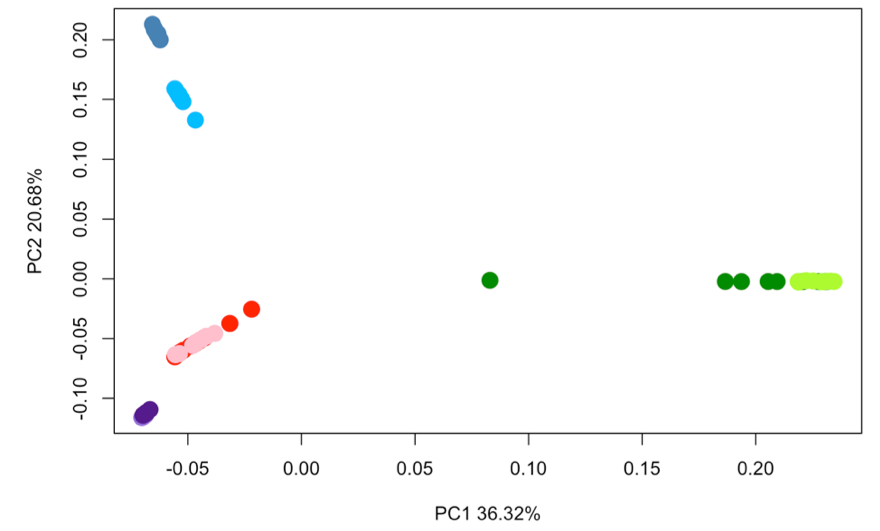
**

**Extended Data Figure 2:** PCA plot based on approximately 21 million SNPs for all four species (and 8 subspecies) combined. Axes titles include the percent variation explained by each axis of the PCA. Colors correspond to populations: savannah sparrows (dark blue – coastal, light blue – interior); Nelson’s sparrows (dark green – coastal, light green – interior); song sparrows (red – coastal, pink – interior); swamp sparrows (dark purple – coastal, light purple – interior).


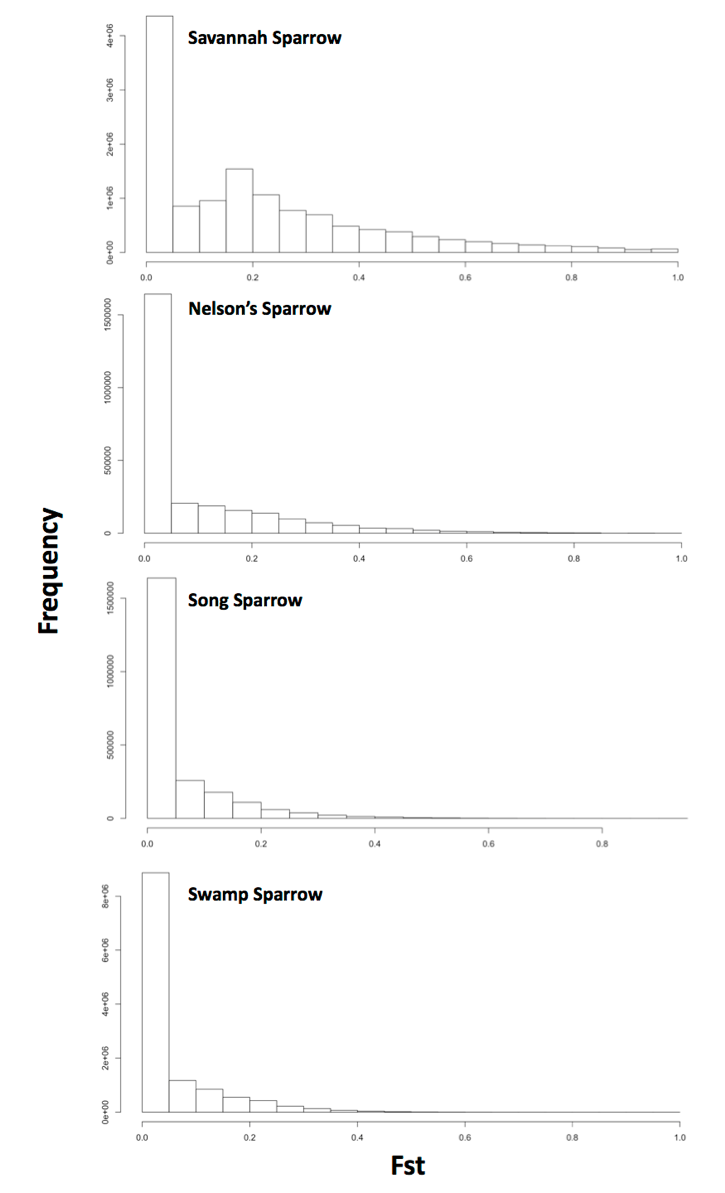


**Extended Data Figure 3:** Histograms of *F*_ST_ estimates for individual SNPs for each of the four species comparisons.

**
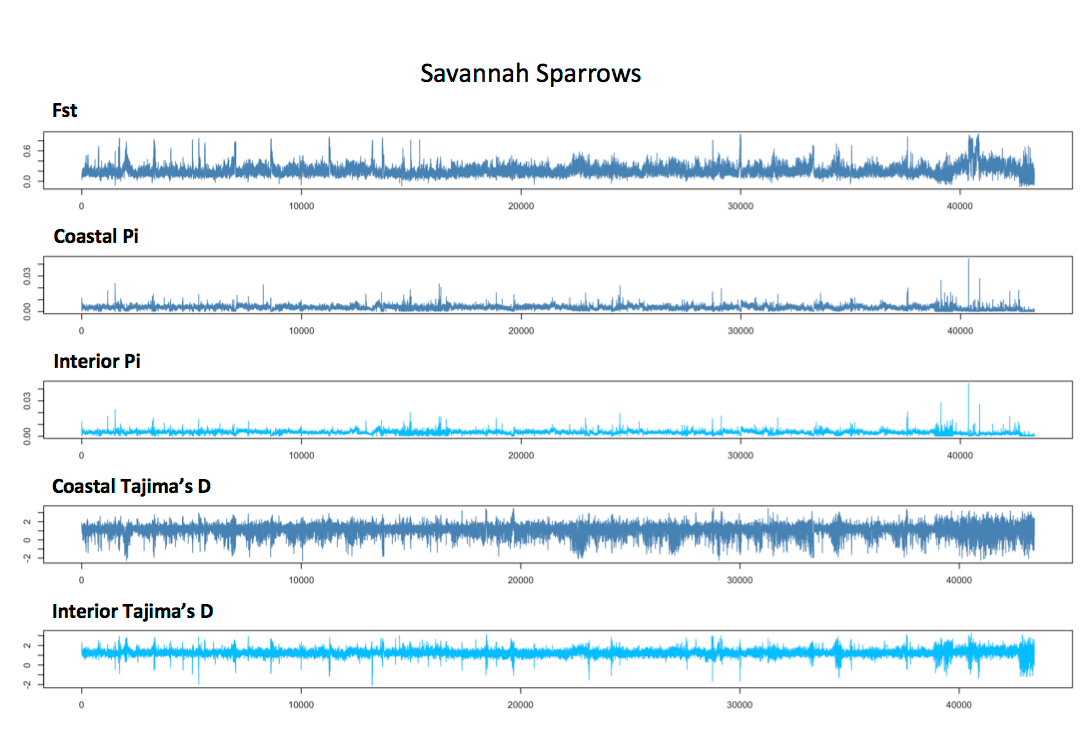
**

**Extended Data Figure 4:** Descriptive statistics for coastal and interior populations of savannah sparrows

**
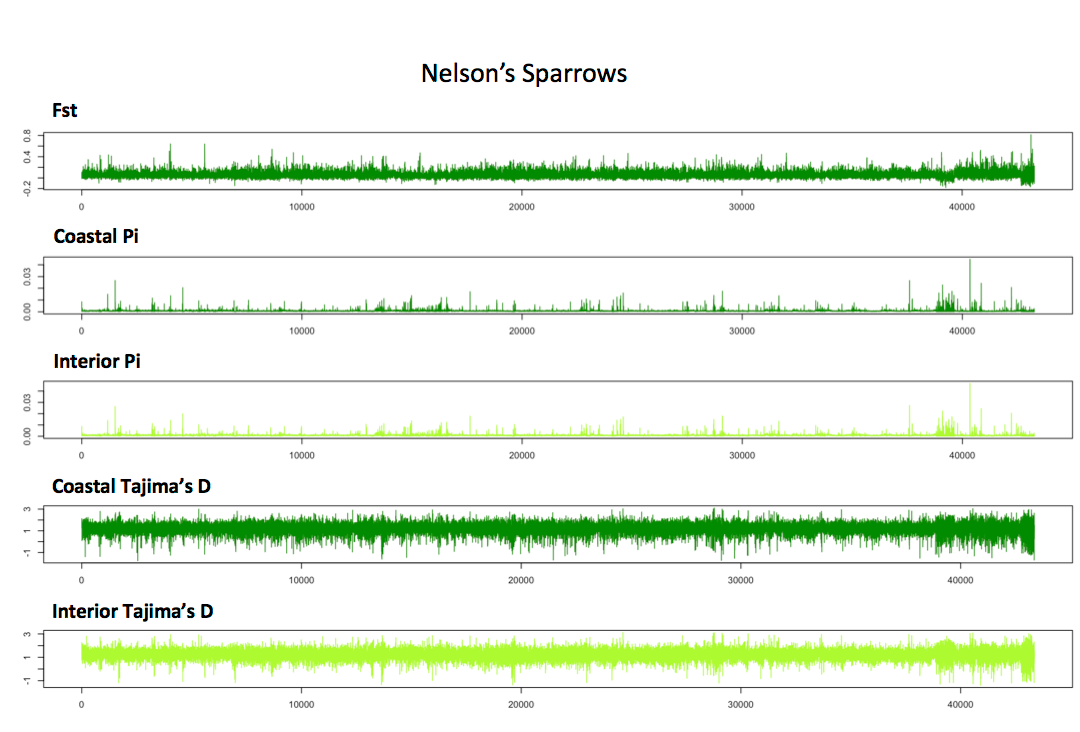
**

**Extended Data Figure 5:** Descriptive statistics for coastal and interior populations of Nelson’s sparrows


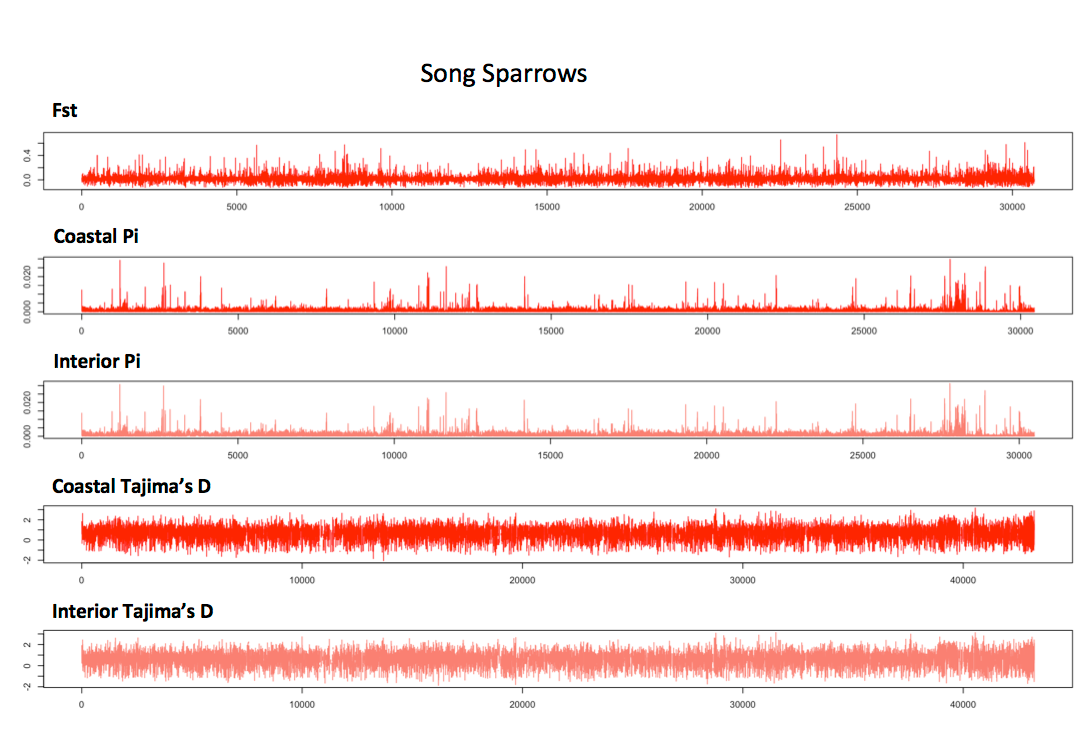


**Extended Data Figure 6:** Descriptive statistics for coastal and interior populations of song sparrows

**
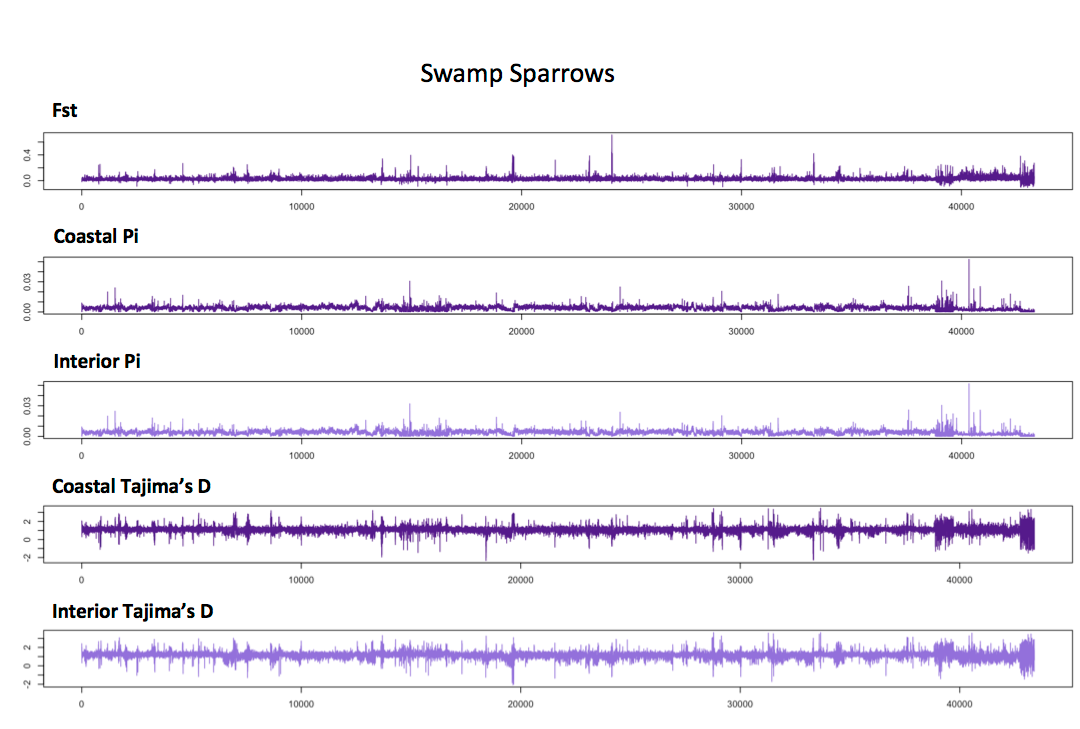
**

**Extended Data Figure 7:** Descriptive statistics for coastal and interior populations of swamp sparrows

**Extended Data Figure 8:** Simulated *F*_ST_ distributions for each species based on neutral demographic history inferred with ∂a∂i. Dashed lines represent the *F*_ST_ cutoff of five standard deviations greater than the mean used to identify outlier regions. Gray dashed line refers to the cutoff based on the simulated dataset and the red line represents empirical *F*_ST_ cutoff used to identify outlier regions in genome scan analyses.

**Extended Data Figure 9:** Distribution of 95^th^ percentiles of the *F*_ST_ distribution from 1000 simulated SNP datasets. For each of the 1000 simulations demographic parameters were randomly sampled from a uniform distribution bounded by the 95% CIs for parameter estimates. Gray dashed line signifies the 99^th^ percentile of the plotted distribution and red dashed line represents the empirical *F*_ST_ cutoff (5 standard deviations greater than mean) used to identify outlier regions.

**Extended Data Figure 10:** Distribution of 99^th^ percentiles of the *F*_ST_ distribution from 1000 simulated SNP datasets. For each of the 1000 simulations demographic parameters were randomly sampled from a uniform distribution bounded by the 95% CIs for parameter estimates. Gray dashed line signifies the 99^th^ percentile of the plotted distribution and red dashed line represents the empirical *F*_ST_ cutoff (5 standard deviations greater than mean) used to identify outlier regions.


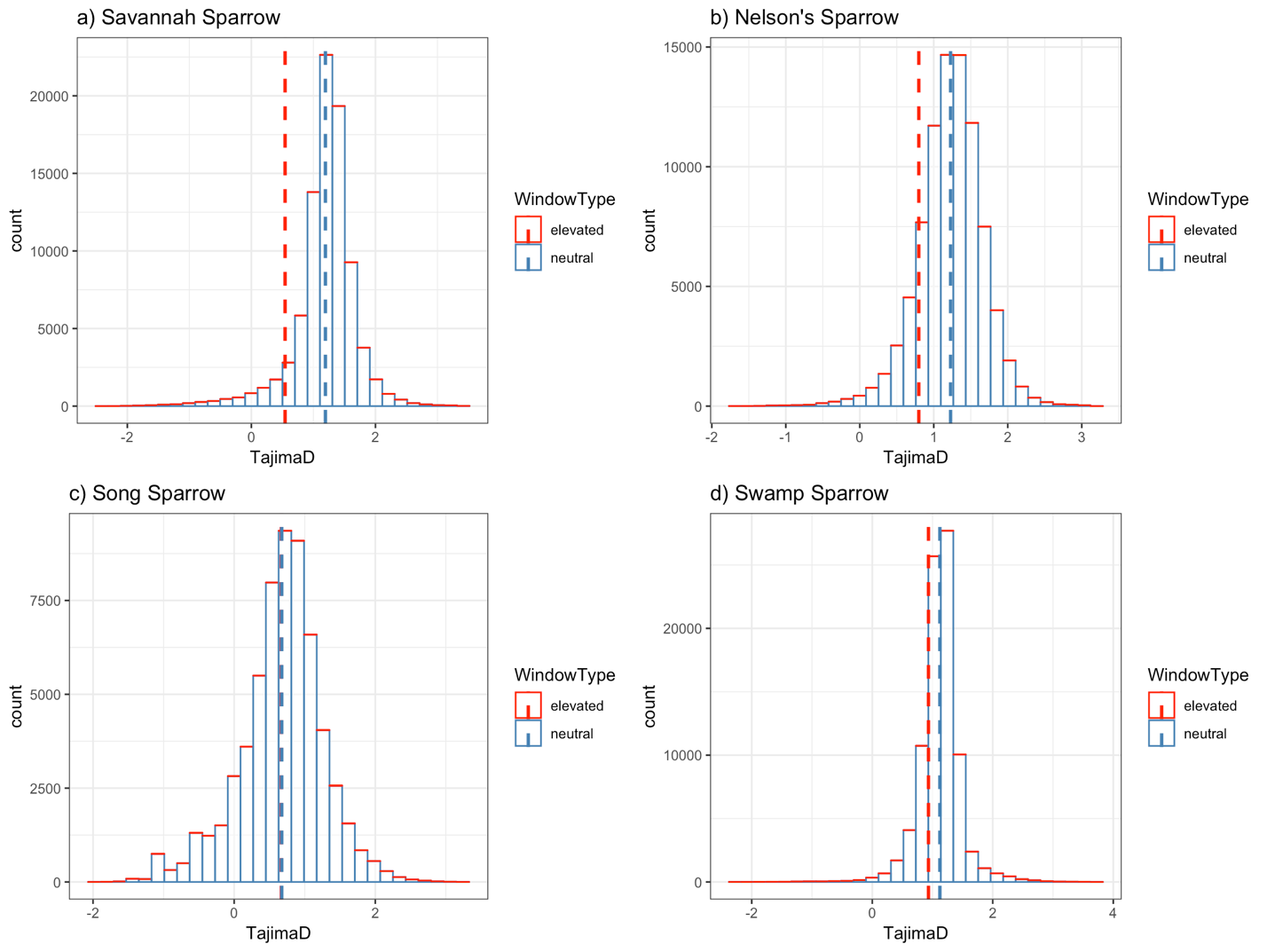


**Extended Figure 11:** Distribution of Tajima’s D estimates for each species comparison for elevated (red) and neutral (genome-wide; blue) windows. The mean estimate for each group is depicted by the dashed line.


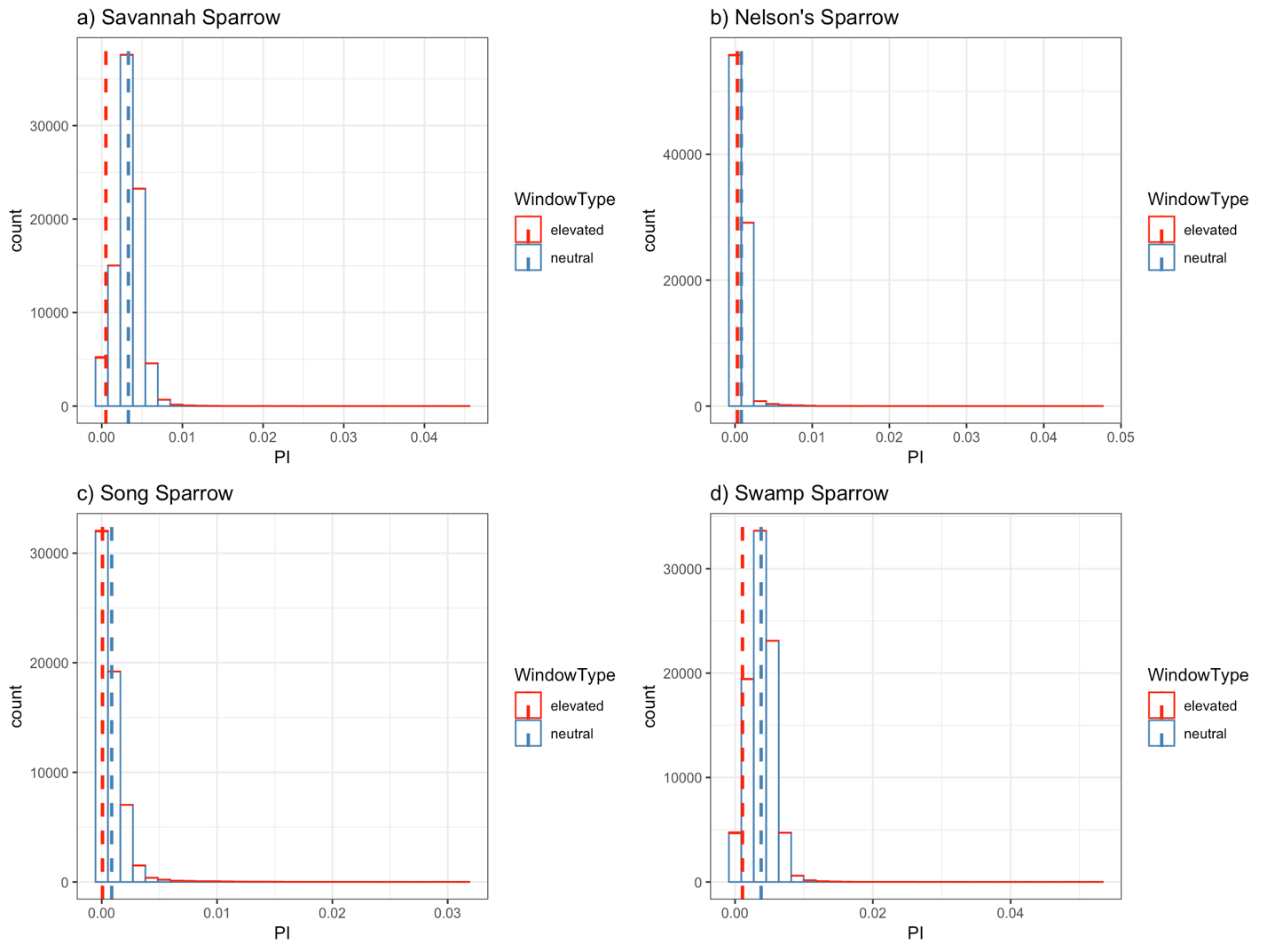


**Extended Figure 12:** Distribution of nucleotide diversity estimates for each species comparison for elevated (red) and neutral (genome-wide; blue) windows. The mean estimate for each group is depicted by the dashed line.

**
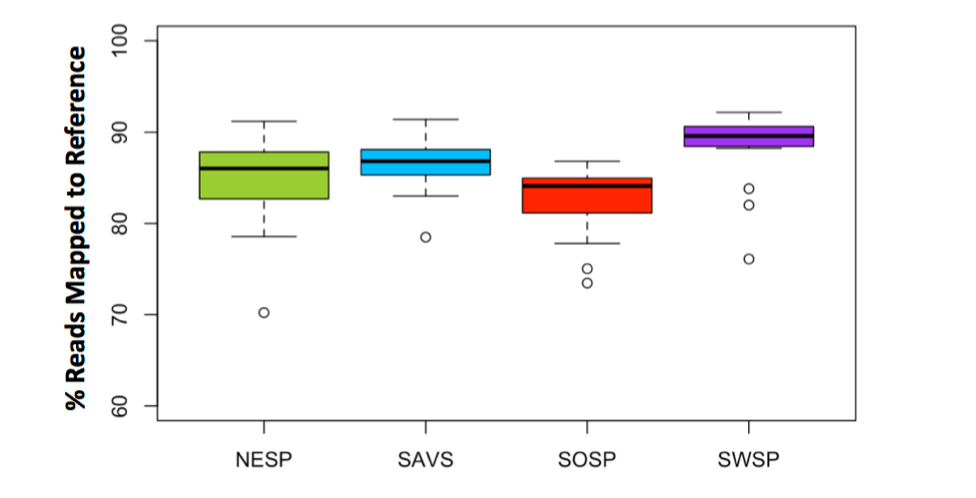
**

**Extended Data Figure 13:** Boxplots depicting the percentage of reads mapped to the swamp sparrow reference genome (color coded by species). Bolded line represents the median and whisker lines represent the 25^th^ and 75^th^ percentile.

**Extended Figure 14:** Different demographic models fit to joint site frequency spectrum of upland (light gray) and tidal marsh populations (dark gray). We fit models with and without migration (*m1, m2)* between the two populations for all population size change models. Parameters estimated are labeled on each model. Population sizes (Ne) during different events are labeled: current (Ne-current); ancestral (Ne-anc); bottleneck (Ne-bot); and contemporary population size (Ne-fresh/salt). Labeling for time parameters: beginning of growth period (Tgrow); bottleneck start (Tbot); bottleneck end (Trec); divergence time (Tsplit); and merger time (Tmerge).

**Supplementary Notes & Results:**

**Demographic Modeling**

The best supported demographic model for savannah sparrows was an isolation with migration model with a bottleneck occurring in the salt marsh population following divergence (LL= -4812.24; Extended Data Table 2) 445,959 ya (CI: 438501-455886; Extended Data; Fig. 1). Bidirectional migration between populations occurred following divergence with greater migration from upland population into salt marsh (m=0.013). Effective population sizes were small for both upland and salt marsh populations with the salt marsh population showing evidence for an extreme bottleneck following divergence; however, there was substantial uncertainty around this estimate. These divergence times are consistent with previous estimates of divergence times between salt marsh and upland savannah sparrows based on RADseq data (~480,000 kya; Benham & Cheviron 2019). Bidirectional migration was also found for the RADseq data set, but estimated population sizes were significantly larger, which is likely due to the greater population sampling of that study (Benham & Cheviron 2019).

A similar demographic model was the best fit for Nelson's sparrow (LL= -1852.91; Extended Data Table 2), with a more recent divergence between upland and salt marsh populations 10,197 ya (CI: 9089-11812 ya; Extended Data; Fig. 2). Upland and salt marsh populations were both characterized to have relatively small effective population sizes (Ne<1000), and migration rate was roughly symmetrical and comparable to other species pairs despite being the most geographically separate pair of populations (North Dakota and Nova Scotia). These results support a previously hypothesized Holocene colonization of tidal marshes in the Canadian Maritime Provinces and northeast United States by interior Nelson's sparrow (Greenlaw 1993; Rising & Avise 1993).

The best-fit model for song sparrows also points to a history of isolation with migration; however, both upland and tidal marshes show evidence for a bottleneck (LL = -1020.4; Extended Data Table 2). The two populations split 12,919 ya (CI:; Extended Data; Fig. 3). Population sizes were found to be larger for the upland population, and a pattern of asymmetric gene flow was found with greater migration from the upland population into the salt marsh population. Contemporary salt marshes in the San Francisco Bay area are estimated to have formed between 6,000-2,000 ya (Atwater 1979; Malamud-Roam et al. 2006), shortly after the predicted divergence date between the sampled upland and tidal marsh populations. Previous microsatellite work found that *M. m. pusillula* was weakly divergent from upland populations, but was still the most divergent salt marsh song sparrow subspecies in the Bay Area (Chan & Arcese 2002). The same study also found fairly high levels of gene flow among these populations, consistent with our results.

The best supported demographic model for swamp sparrows was also an isolation with migration model with a bottleneck occurring in the upland population following divergence (LL= -1449.79; Extended Data Table 2) of 35,626 ya (CI: 35270-36139) between upland and salt marsh populations (Extended Data; Fig. 4). Upland and salt marsh populations were both characterized to have the largest effective populations sizes of any species. Migration rates were roughly symmetrical.

**Supplementary Tables:**

**Extended Data Table 1:** Information and Sampling locations for individual sparrows analyzed in this study

| **Individual ID** | **Species** | **Sex** | **Population** | **Site** |
| --- | --- | --- | --- | --- |
| 2391-71742 | *A. n. subvirgatus* | Male | Coastal | Yarmouth, Nova Scotia |
| 2391-71759 | *A. n. subvirgatus* | Male | Coastal | Yarmouth, Nova Scotia |
| 2391-71763 | *A. n. subvirgatus* | Male | Coastal | Yarmouth, Nova Scotia |
| 2391-71767 | *A. n. subvirgatus* | Male | Coastal | Wolfville, Nova Scotia |
| 2391-71768 | *A. n. subvirgatus* | Male | Coastal | Wolfville, Nova Scotia |
| 2391-71769 | *A. n. subvirgatus* | Male | Coastal | Wolfville, Nova Scotia |
| 2391-71748 | *A. n. subvirgatus* | Male | Coastal | Yarmouth, Nova Scotia |
| 2391-71749 | *A. n. subvirgatus* | Male | Coastal | Yarmouth, Nova Scotia |
| 2391-71771 | *A. n. subvirgatus* | Male | Coastal | Wolfville, Nova Scotia |
| 1771-02770 | *A. n. nelsoni* | Male | Interior | Grand Forks - North Dakota |
| 1771-02777 | *A. n. nelsoni* | Male | Interior | Grand Forks - North Dakota |
| 1771-02774 | *A. n. nelsoni* | Male | Interior | Grand Forks - North Dakota |
| 2321-42835 | *A. n. nelsoni* | Male | Interior | Grand Forks - North Dakota |
| 1771-02778 | *A. n. nelsoni* | Male | Interior | Jay Clark Salyer - North Dakota |
| 1771-02782 | *A. n. nelsoni* | Male | Interior | Jay Clark Salyer - North Dakota |
| 1771-02784 | *A. n. nelsoni* | Male | Interior | Jay Clark Salyer - North Dakota |
| 1771-02785 | *A. n. nelsoni* | Male | Interior | Jay Clark Salyer - North Dakota |
| 2321-03301 | *P. s. beldingi* | Male | Coastal | Upper Newport Bay - California |
| 2321-03302 | *P. s. beldingi* | Male | Coastal | Upper Newport Bay - California |
| 2321-03303 | *P. s. beldingi* | Male | Coastal | Upper Newport Bay - California |
| 2321-03304 | *P. s. beldingi* | Male | Coastal | Upper Newport Bay - California |
| 2321-03305 | *P. s. beldingi* | Male | Coastal | Upper Newport Bay - California |
| 2321-03306 | *P. s. beldingi* | Male | Coastal | Upper Newport Bay - California |
| 2321-03307 | *P. s. beldingi* | Male | Coastal | Upper Newport Bay - California |
| 2321-03308 | *P. s. beldingi* | Male | Coastal | Upper Newport Bay - California |
| 2321-03309 | *P. s. beldingi* | Male | Coastal | Upper Newport Bay - California |
| 2321-03310 | *P. s. beldingi* | Male | Coastal | Upper Newport Bay - California |
| FMNH 499944 | *P. s. nevadensis* | Male | Interior | Lassen National Forest - California |
| FMNH 499945 | *P. s. nevadensis* | Male | Interior | Lassen National Forest - California |
| FMNH 499946 | *P. s. nevadensis* | Male | Interior | Lassen National Forest - California |
| FMNH 499947 | *P. s. nevadensis* | Male | Interior | Lassen National Forest - California |
| FMNH 499948 | *P. s. nevadensis* | Male | Interior | Lassen National Forest - California |
| FMNH 499949 | *P. s. nevadensis* | Male | Interior | Lassen National Forest - California |
| FMNH 499951 | *P. s. nevadensis* | Male | Interior | San Bernardino National Forest - California |
| FMNH 499952 | *P. s. nevadensis* | Male | Interior | San Bernardino National Forest - California |
| FMNH 499953 | *P. s. nevadensis* | Male | Interior | San Bernardino National Forest - California |
| FMNH 499954 | *P. s. nevadensis* | Male | Interior | San Bernardino National Forest - California |
| 9971 | *M. m. pusillula* | Male | Coastal | Palo Alto Baylands Nature Preserve - California |
| 9974 | *M. m. pusillula* | Female | Coastal | Palo Alto Baylands Nature Preserve - California |
| 9958 | *M. m. pusillula* | Male | Coastal | Palo Alto Baylands Nature Preserve - California |
| 9968 | *M. m. pusillula* | Male | Coastal | Palo Alto Baylands Nature Preserve - California |
| 9963 | *M. m. pusillula* | Male | Coastal | Palo Alto Baylands Nature Preserve - California |
| 9975 | *M. m. pusillula* | Male | Coastal | Palo Alto Baylands Nature Preserve - California |
| 9959 | *M. m. pusillula* | Male | Coastal | Palo Alto Baylands Nature Preserve - California |
| 9965 | *M. m. pusillula* | Male | Coastal | Palo Alto Baylands Nature Preserve - California |
| 9967 | *M. m. pusillula* | Male | Coastal | Palo Alto Baylands Nature Preserve - California |
| 9964 | *M. m. pusillula* | Female | Coastal | Palo Alto Baylands Nature Preserve - California |
| 99191 | *M. m. gouldii* | Male | Interior | Mark's Marsh, Tomales Bay - California |
| 99195 | *M. m. gouldii* | Male | Interior | Mark's Marsh, Tomales Bay - California |
| 99194 | *M. m. gouldii* | Male | Interior | Mark's Marsh, Tomales Bay - California |
| 99326 | *M. m. gouldii* | Male | Interior | Mark's Marsh, Tomales Bay - California |
| 99183 | *M. m. gouldii* | Male | Interior | Mark's Marsh, Tomales Bay - California |
| 99185 | *M. m. gouldii* | Male | Interior | Mark's Marsh, Tomales Bay - California |
| 99186 | *M. m. gouldii* | Male | Interior | Mark's Marsh, Tomales Bay - California |
| 99217 | *M. m. gouldii* | Male | Interior | Mark's Marsh, Tomales Bay - California |
| 99193 | *M. m. gouldii* | Male | Interior | Mark's Marsh, Tomales Bay - California |
| 99192 | *M. m. gouldii* | Female | Interior | Mark's Marsh, Tomales Bay - California |
| BT4420 | *M. g. nigrescens* | Male | Coastal | Fishing Bay – Maryland |
| BT4422 | *M. g. nigrescens* | Male | Coastal | Fishing Bay – Maryland |
| 1691-39703 | *M. g. nigrescens* | Male | Coastal | Fishing Bay – Maryland |
| 1691-39704 | *M. g. nigrescens* | Male | Coastal | Fishing Bay – Maryland |
| 1691-39705 | *M. g. nigrescens* | Male | Coastal | Fishing Bay – Maryland |
| 1691-39706 | *M. g. nigrescens* | Male | Coastal | Fishing Bay – Maryland |
| 1691-39708 | *M. g. nigrescens* | Male | Coastal | Fishing Bay – Maryland |
| 1361-27299 | *M. g. nigrescens* | Male | Coastal | Port Mahone - Delaware |
| 1361-27300 | *M. g. nigrescens* | Male | Coastal | Port Mahone - Delaware |
| 1361-27284 | *M. g. nigrescens* | Male | Coastal | Port Mahone - Delaware |
| 1361-27210 | *M. g. nigrescens* | Male | Coastal | Port Mahone - Delaware |
| BT4113 | *M. g. georgiana* | Male | Interior | Tompkins County – New York |
| BT4112 | *M. g. georgiana* | Male | Interior | Tompkins County – New York |
| BT1872 | *M. g. georgiana* | Male | Interior | Tompkins County – New York |
| BT2254 | *M. g. georgiana* | Male | Interior | Tompkins County – New York |
| BT3060 | *M. g. georgiana* | Male | Interior | Tompkins County – New York |
| BT4418 | *M. g. georgiana* | Male | Interior | Tompkins County – New York |
| BT4417 | *M. g. georgiana* | Male | Interior | Tompkins County – New York |
| 1361-27242 | *M. g. georgiana* | Male | Interior | Erie County - Pennsylvania |
| 1361-27241 | *M. g. georgiana* | Male | Interior | Erie County - Pennsylvania |
| 1361-27223 | *M. g. georgiana* | Male | Interior | Garrett County - Maryland |
| 1361-27226 | *M. g. georgiana* | Male | Interior | Garrett County - Maryland |
| BT4079 | *M. g. georgiana* | Male | Interior | Hamilton County – New York |

**Extended Data Table 2:** Average observed heterozygosity, nucleotide diversity, and Tajima’s D for coastal and interior populations of savannah, Nelson’s, song, and swamp sparrows

| **Species** | **Population** | **Average Observed Heterozygosity** | **Average Nucleotide Diversity** | **Average Tajima’s D** |  |  |  |
| --- | --- | --- | --- | --- | --- | --- | --- |
| savannah sparrow | Coastal | 0.2195 | 0.0034 | 1.119464 |  |  |  |
| savannah sparrow | Interior | 0.1947 | 0.0032 | 1.265654 |  |  |  |
| Nelson's sparrow | Coastal | 0.2604 | 0.0008 | 1.218415 |  |  |  |
| Nelson's sparrow | Interior | 0.2884 | 0.0008 | 1.234479 |  |  |  |
| song sparrow | Coastal | 0.2232 | 0.0008 | 0.7073458 |  |  |  |
| song sparrow | Interior | 0.2458 | 0.0009 | 0.6420787 |  |  |  |
| swamp sparrow | Coastal | 0.2877 | 0.0038 | 1.086498 |  |  |  |
| swamp sparrow | Interior | 0.2777 | 0.0037 | 1.158988 |  |  |  |

**Extended Data Table 3:** Parameter estimates for the best-fit demographic model from ∂a∂i. Table includes the best-fit demographic model based on log-likelihoods (LL). Nref is the ancestral population size, fresh refers to upland population and salt the tidal marsh population. Bot is bottleneck and rec post-bottleneck recovery.

|  | Savannah | Nelson’s | Song | Swamp |
| --- | --- | --- | --- | --- |
| best-fit model | IM bottleneck salt | IM bottleneck salt | IM bottleneck both | IM bottleneck fresh |
| LL | -4812.24 | -1852.91 | -1376.21 | -1449.79 |
| Theta (4Neµ) | 1235.17 (1230.4-1239.9) | 256.42 (251.42-261.42) | 225.16 (62.73-362.31) | 913.08 |
| Nref | 396 (395-398) | 82 (81-84) | 72 (20-116) | 293 (293-293) |
| Ne_fresh | 158 (1-1126) | 940 (315-1590) | 547 (1-3700) | 6129 (6075-6184) |
| Ne_salt | 5 (1-1422) | 64 (1-1995) | 6409 (937-17217) | 3681 (3411-3952) |
| Ne_bot_fresh | NA | NA | 975 (1-16020) | 10813 (10799-10827) |
| Ne_rec_fresh | NA | NA | 179 (40-350) | 10170 (10139-10202) |
| Ne_bot_salt | 8036 (5986-10101) | 40 (1-604) | 50 (6-134) | NA |
| Ne_rec_salt | 419 (1-1972) | 150 (135-164) | 324 (45-785) | NA |
| Time split | 445959 (438501-455886) | 10197 (9089-11812) | 12919 (10-47035) | 35626 (35270-36139) |
| Time_bot_fresh | NA | NA | 260 (9-6463) | 576 (338-970) |
| Time_rec_fresh | NA | NA | 253 (8-6371) | 75 (1-306) |
| Time_bot_salt | 24962 (21643-30743) | 104 (1-683) | 77 (7-165) | NA |
| Time_rec_salt | 221 (1-2881) | 25 (1-174) | 28 (4-66) | NA |
| m1 (salt–>fresh) | 0.013 (0.0052-0.0207) | 0.0442 (0.0177-0.0697) | 0.4732 (0.312-1.44) | 0.0056 (0.0044-0.0067) |
| m2 (fresh–>salt) | 0.0000153 (0-0.006) | 0.0771 (0.0656-0.0881) | 0.00015 (0-0.0005) | 0.0079 (0.0074-0.0084) |

**Extended Data Table 4:** List of shared candidate genes in two or three species pairs identified through a comparative genomics approach.

| **Candidate Gene** | **Sparrow species exhibiting elevated divergence** | **Function** | **Putative Adaptive Function** |
| --- | --- | --- | --- |
| YTHDF2 | savannah, swamp | Response to heat shock | Heat stress, thermal regulation |
| RBM39 | Nelson's, savannah, swamp | RNA binding protein; knockdown of RBM39 produced strong enhancement of BMP4 in cell cultures | Linked to increased bill size in marsh birds (heat response, thermal regulation |
| C9 | song, swamp | Innate and adaptive immune response (bacteria) | Response to increased bacteria in tidal environments |
| ROMO1 | Nelson's, savannah, swamp | Defense response to bacteria | Response to increased bacteria in tidal environments |
| DAB2 | song, swamp | Vesicle transport and Wnt signaling | Osmotic stress, melanism |
| MAPK8IP3 | song, swamp | Vesicle transport through interactions with JNK-signaling components | Osmotic stress |
| PPIP5K1 | Nelson's, swamp | Involved in vesicle trafficking, activated when cells are exposed to hyperosmotic stress | Osmotic stress |
| PPIP5K2 | Nelson's, savannah | Involved in vesicle trafficking | Osmotic stress |
| RBM12 | Nelson's Sparrow, Swamp Sparrow | RNA binding protein; downregulated in cell cultures exposed to sodium chloride-Induced osmotic stress | Osmotic stress |
| SLC41A2 | Nelson's, savannah, swamp | Transmembrane transport; magnesium/salt exchange (magnesium being the second most abundant cation in salt water) | Osmotic stress |
| TAF12 | savannah, swamp | Transcriptional activator; linked to stress response in plants, differentially expressed in freshwater versus marine sticklebacks | Osmotic stress |
| TMEM161B | Nelson's, savannah | Transmembrane protein | Osmotic stress |
| ARHGAP5 | swamp, Nelson's | Cell adhesion, Rho protein signal transduction. Rho GTP-ases regulate cytoskeleton, which is important in reinforcing cell structure to withstand challenges of hyperosmotic stress | Osmotic stress |
| DLC1 | song, swamp, savannah | Rho GTP-ase activating protein; plays critical role in biological processes such as cell migration. Rho GTP-ases regulate cytoskeleton, which is important in reinforcing cell structure to withstand challenges of hyperosmotic stress | Osmotic stress |
| EPB41L4A | savannah, swamp | Structural constituent of cytoskeleton | Osmotic stress |
| PAM | Nelson's, savannah | Multiple biological processes, including regulation of actin cytoskeleton organization | Osmotic stress |
| AASDH | savannah, swamp | Fatty acid metabolic process | unknown |
| ABLIM3 | Nelson's, song | Scaffold protein; differentially expressed in freshwater versus marine sticklebacks | unknown |
| CCNH | Nelson's, savannah | Involved in cell cycle control and in RNA transcription by RNA polymerase II | unknown |
| FAR2 | song, savannah | Lipid metabolic process | unknown |
| GIN1 | Nelson's, savannah | DNA integration | unknown |
| GLYR1 | song, swamp | DNA binding, histone binding | unknown |
| GMEB1 | savannah, swamp | Increases sensitivity to low concentrations of glucocorticoids | unknown |
| KIAA1456 | song, savannah, swamp | May modify wobble uridines in specific arginine and glutamic acid tRNAs | unknown |
| LAYN | Nelson's, savannah | Receptor for hyaluronate | unknown |
| LONRF1 | savannah, swamp | peptidase N-terminal domain and RING finger protein 1 | unknown |
| MRPS34 | song, swamp | Required for mitochondrial translation | unknown |
| NDRG3 | savannah, swamp | Cell differentiation, spermatogenesis | unknown |
| PHF20 | savannah, swamp | Histone acetylation | unknown |
| STARD4 | savannah, swamp | Cholesterol import | unknown |
| TRNAU1AP | savannah, swamp | Involved in the early steps of selenocysteine biosynthesis and tRNA | unknown |
| TUBGCP4 | Nelson's, swamp | Gamma-tubulin complex is necessary for microtubule nucleation at the centrosome | unknown |
| WASHC4 | savannah, swamp | Endosomal transport | unknown |

**Extended Data Table 5:** Candidate genes identified through whole genome comparisons of freshwater and salt water populations of Savannah Sparrows. Genes are linked to potential adaptive functions, including osmoregulatory (OR), kidney development (KD), circadian rhythm (CR), bill morphology (BM), melanin pathways (MP), or response to heat stress (HS). Window-based Tajima’s D and pi estimates are presented for both coastal and interior populations. The strongest candidates (elevated *F*_ST_ and negative Tajima’s D in one population) are highlighted in grey, genes that are shared with at least one other species pair are highlighted in blue.

| **Chromosome** | **Position Start** | **Gene ID** | **Putative Adaptive Function** | **Tajima's D Coastal** | **Tajima's D Interior** | **Pi Coastal** | **Pi Interior** |
| --- | --- | --- | --- | --- | --- | --- | --- |
| Chromosome 1 | 104,225,001 | ST3GAL6 |  | 1.68444 | 0.724557 | 0.000472872 | 6.19E-05 |
| Chromosome 1 | 104,600,001 | DCBLD2 |  | 1.08965 | 0.528181 | 0.000669489 | 0.000195182 |
| Chromosome 12 | 150,001 | BICD2 |  | -0.675768 | 1.55665 | 0.000224219 | 0.00039542 |
| Chromosome 12 | 250,001 | FGD3 | OR | -1.209 | 1.05569 | 0.00026233 | 0.00148802 |
| Chromosome 12 | 300,001 | SUSD3 |  | -0.185643 | -0.474942 | 0.00144887 | 0.00120245 |
| Chromosome 12 | 300,001 | CARD19 |  | -0.185643 | -0.474942 | 0.00144887 | 0.00120245 |
| Chromosome 12 | 300,001 | NINJ1 |  | -0.185643 | -0.474942 | 0.00144887 | 0.00120245 |
| Chromosome 12 | 450,001 | WNK2 | OR | 0.378483 | 0.282095 | 0.00026569 | 0.00161605 |
| Chromosome 12 | 475,001 | FAM120A |  | 1.26083 | -0.882471 | 0.000271379 | 0.000782452 |
| Chromosome 13 | 1,850,001 | NEURL1B |  | 1.66499 | 1.27599 | 0.00451249 | 0.00402106 |
| Chromosome 13 | 2,350,001 | ERGIC1 |  | 1.18314 | 1.54518 | 0.00368028 | 0.00380194 |
| Chromosome 13 | 2,350,001 | CREBRF |  | 1.18314 | 1.54518 | 0.00368028 | 0.00380194 |
| Chromosome 17 | 10,750,001 | DPP7 |  | -0.37821 | 0.337122 | 0.000130531 | 0.000410574 |
| Chromosome 17 | 10,750,001 | MAN1B1 |  | -0.37821 | 0.337122 | 0.000130531 | 0.000410574 |
| Chromosome 17 | 10,750,001 | UAP1L1 |  | -0.37821 | 0.337122 | 0.000130531 | 0.000410574 |
| Chromosome 17 | 10,750,001 | SAPCD2 |  | -0.37821 | 0.337122 | 0.000130531 | 0.000410574 |
| Chromosome 17 | 10,875,001 | ENTPD8 | OR | 0.441185 | -0.595092 | 0.000229277 | 0.000616148 |
| Chromosome 17 | 10,875,001 | ABCA2 |  | 0.441185 | -0.595092 | 0.000229277 | 0.000616148 |
| Chromosome 17 | 10,875,001 | FUT7 |  | 0.441185 | -0.595092 | 0.000229277 | 0.000616148 |
| Chromosome 1A | 22,875,001 | KCNA1 | OR | -0.260973 | 0.467716 | 0.000213477 | 0.00044936 |
| Chromosome 1A | 23,850,001 | FAR2 |  | 1.82942 | 0.736856 | 0.000139158 | 7.37E-05 |
| Chromosome 1A | 63,725,001 | TDG |  | 1.33385 | 0.470555 | 0.000127162 | 9.64E-05 |
| Chromosome 1A | 63,725,001 | GLT8D2 |  | 1.33385 | 0.470555 | 0.000127162 | 9.64E-05 |
| Chromosome 1A | 63,725,001 | HCFC2 |  | 1.33385 | 0.470555 | 0.000127162 | 9.64E-05 |
| Chromosome 1A | 63,725,001 | NFYB |  | 1.33385 | 0.470555 | 0.000127162 | 9.64E-05 |
| Chromosome 1A | 64,125,001 | SLC41A2 | OR | 1.06948 | -0.060869 | 4.74E-05 | 0.000107374 |
| Chromosome 1A | 64,125,001 | WASHC4 |  | 1.06948 | -0.060869 | 4.74E-05 | 0.000107374 |
| Chromosome 20 | 200,001 | RBM1 | OR | -0.945303 | 1.33096 | 0.000135583 | 0.000342136 |
| Chromosome 20 | 200,001 | ROMO1 |  | -0.945303 | 1.33096 | 0.000135583 | 0.000342136 |
| Chromosome 20 | 200,001 | RBM39 | BM | -0.945303 | 1.33096 | 0.000135583 | 0.000342136 |
| Chromosome 20 | 275,001 | PHF20 |  | -0.699021 | 1.04888 | 5.24E-05 | 0.00014738 |
| Chromosome 20 | 400,001 | NDRG3 |  | -1.52018 | 0.634286 | 4.02E-05 | 0.000255594 |
| Chromosome 20 | 450,001 | RALGAPB |  | -1.52018 | 0.634286 | 4.88E-05 | 0.00013011 |
| Chromosome 23 | 75,001 | YTHDF2 |  | -0.852234 | 0.234826 | 0.000236434 | 0.000745672 |
| Chromosome 23 | 75,001 | GMEB1 |  | -0.852234 | 0.234826 | 0.000236434 | 0.000745672 |
| Chromosome 23 | 75,001 | TAF12 |  | -0.852234 | 0.234826 | 0.000236434 | 0.000745672 |
| Chromosome 23 | 75,001 | TRNAU1AP |  | -0.852234 | 0.234826 | 0.000236434 | 0.000745672 |
| Chromosome 24 | 1,900,001 | LAYN |  | 0.0674041 | NA | 9.47E-06 | NA |
| Chromosome 4 | 8,750,001 | UGT8 |  | 0.318133 | 1.58425 | 0.000123794 | 0.000487641 |
| Chromosome 4 | 8,900,001 | ARSJ |  | 0.637322 | 0.574027 | 0.00017832 | 0.000412086 |
| Chromosome 4 | 39,925,001 | KDR | OR | -0.851744 | -1.62062 | 0.000303413 | 0.00034683 |
| Chromosome 4 | 39,975,001 | SRD5A3 |  | 1.63526 | -0.821612 | 0.000340231 | 0.000295183 |
| Chromosome 4 | 40,000,001 | TMEM165 | OR | 0.758374 | -1.13799 | 0.000121059 | 0.000286353 |
| Chromosome 4 | 40,325,001 | KIAA1211 |  | 2.07641 | 0.801992 | 0.000223372 | 8.21E-05 |
| Chromosome 4 | 40,350,001 | AASDH |  | 1.28356 | 1.25267 | 8.61E-05 | 9.77E-05 |
| Chromosome 4 | 40,475,001 | LONRF1 |  | -1.40617 | 0.491256 | 4.29E-05 | 4.25E-05 |
| Chromosome 4 | 40,600,001 | KIAA1456 |  | 0.969543 | 0.633213 | 0.000134529 | 6.15E-05 |
| Chromosome 4 | 40,600,001 | DLC1 | OR | 0.969543 | 0.633213 | 0.000134529 | 6.15E-05 |
| Z chromosome | 19,075,001 | EFNA5 |  | 1.28441 | 1.50884 | 0.000175595 | 0.000152214 |
| Z chromosome | 20,575,001 | NUDT12 |  | -1.0437 | 0.770977 | 0.000617962 | 0.000309509 |
| Z chromosome | 20,700,001 | PPIP5K2 | OR | -0.906703 | 1.36364 | 0.00058253 | 0.000124426 |
| Z chromosome | 20,725,001 | GIN1 |  | -0.889534 | -0.0271862 | 0.000703119 | 0.000137477 |
| Z chromosome | 20,775,001 | PAM |  | -0.791917 | 0.9009 | 0.000862941 | 0.000196639 |
| Z chromosome | 22,250,001 | WDR36 |  | 0.793403 | 2.06235 | 0.000299205 | 0.000611056 |
| Z chromosome | 22,350,001 | STARD4 |  | 1.41955 | 1.84663 | 0.000414822 | 0.000761989 |
| Z chromosome | 22,625,001 | EPB41L4A | OR | 0.860921 | 1.85407 | 0.00023771 | 0.000544493 |
| Z chromosome | 22,875,001 | APC | OR | 0.0385973 | 1.16621 | 0.00017961 | 0.000937219 |
| Z chromosome | 22,900,001 | SRP19 |  | 0.423873 | 1.49562 | 0.000143169 | 0.000504265 |
| Z chromosome | 22,975,001 | REEP5 |  | 1.06422 | 2.01687 | 0.00028087 | 0.000594617 |
| Z chromosome | 23,025,001 | MCC | OR | 1.53845 | 1.42291 | 0.000289715 | 0.000725839 |
| Z chromosome | 27,600,001 | NR2F1 |  | 0.6902 | 0.874648 | 0.000196664 | 0.000466156 |
| Z chromosome | 28,400,001 | ARRDC3 | HS | 0.833197 | 2.07228 | 0.000213709 | 0.000375418 |
| Z chromosome | 28,700,001 | ADGRV1 | CR | 0.23057 | 1.52686 | 0.000274558 | 0.00126392 |
| Z chromosome | 28,825,001 | LYSMD3 |  | 0.76237 | 0.357244 | 0.000258753 | 0.000548304 |
| Z chromosome | 28,850,001 | MBLAC2 |  | 1.57798 | 0.548469 | 0.000327195 | 0.00056366 |
| Z chromosome | 28,850,001 | CETN3 |  | 1.57798 | 0.548469 | 0.000327195 | 0.00056366 |
| Z chromosome | 29,575,001 | MEF2C |  | 0.308188 | 0.395658 | 0.000190538 | 0.000324867 |
| Z chromosome | 29,825,001 | TMEM161B | OR | -0.947603 | 1.55877 | 0.000889828 | 0.000213066 |
| Z chromosome | 29,975,001 | CCNH |  | -0.874364 | 1.26994 | 0.000523993 | 0.000143798 |
| Z chromosome | 29,975,001 | RASA1 | OR | -0.874364 | 1.26994 | 0.000523993 | 0.000143798 |
| Z chromosome | 30,100,001 | RGMB | BM | -1.00517 | 0.820051 | 0.000501383 | 0.000140846 |

**Extended Data Table 6:** Candidate genes identified through whole genome comparisons of freshwater and salt water populations of Nelson’s Sparrows. Genes are linked to potential adaptive functions, including osmoregulatory (OR), kidney development (KD), circadian rhythm (CR), bill morphology (BM), melanin pathways (MP), or response to heat stress (HS). Window-based Tajima’s D and pi estimates are presented for both coastal and interior populations. The strongest candidates (elevated *F*_ST_ and negative Tajima’s D in one population) are highlighted in grey, genes that are shared with at least one other species pair are highlighted in blue.

| **Chromosome** | **Position Start** | **Gene ID** | **Putative Adaptive Function** | **Tajima's D Coastal** | **Tajima's D Interior** | **Pi Coastal** | **Pi Interior** |
| --- | --- | --- | --- | --- | --- | --- | --- |
| Chromosome 10 | 20,825,001 | PPIP5K1 | OR | -1.36585 | 1.26021 | 0.000163016 | 0.000399406 |
| Chromosome 10 | 20,825,001 | TUBGCP4 |  | -1.36585 | 1.26021 | 0.000163016 | 0.000399406 |
| Chromosome 11 | 9,300,001 | CBFB |  | 0.222033 | 0.598601 | 0.000332738 | 0.000479739 |
| Chromosome 11 | 12,575,001 | SALL1 | KD | 0.88839 | 0.670801 | 0.000637888 | 0.000597081 |
| Chromosome 13 | 1,750,001 | GRPEL2 |  | 1.23854 | 1.12353 | 0.000900605 | 0.000938216 |
| Chromosome 13 | 1,750,001 | AFAP1L1 | OR | 1.23854 | 1.12353 | 0.000900605 | 0.000938216 |
| Chromosome 13 | 1,750,001 | ABLIM3 |  | 1.23854 | 1.12353 | 0.000900605 | 0.000938216 |
| Chromosome 14 | 16,750,001 | KDM8 |  | 1.79952 | 0.183682 | 0.000187677 | 0.000128338 |
| Chromosome 14 | 16,750,001 | NSMCE1 |  | 1.79952 | 0.183682 | 0.000187677 | 0.000128338 |
| Chromosome 14 | 16,775,001 | PDPK1 | OR | 0.185498 | 0.747006 | 0.000187677 | 0.000128338 |
| Chromosome 14 | 16,825,001 | KCTD5 |  | -0.891434 | 1.64364 | 7.27E-05 | 0.000206682 |
| Chromosome 14 | 16,825,001 | TSR3 |  | -0.891434 | 1.64364 | 7.27E-05 | 0.000206682 |
| Chromosome 14 | 16,825,001 | GNPTG |  | -0.891434 | 1.64364 | 7.27E-05 | 0.000206682 |
| Chromosome 15 | 875,001 | SFSWAP |  | -1.25524 | 1.52545 | 0.000136678 | 0.000206349 |
| Chromosome 15 | 900,001 | MMP17 | OR/KD | -0.375539 | 1.46839 | 0.000325721 | 0.000208678 |
| Chromosome 15 | 900,001 | TMEM132D | OR | -0.375539 | 1.46839 | 0.000325721 | 0.000208678 |
| Chromosome 15 | 900,001 | TMEM132C | OR | -0.375539 | 1.46839 | 0.000325721 | 0.000208678 |
| Chromosome 19 | 25,001 | BCL7B |  | 1.64118 | 0.387887 | 0.000445738 | 5.70E-05 |
| Chromosome 19 | 25,001 | TBL2 |  | 1.64118 | 0.387887 | 0.000445738 | 5.70E-05 |
| Chromosome 19 | 25,001 | MLXIPL |  | 1.64118 | 0.387887 | 0.000445738 | 5.70E-05 |
| Chromosome 19 | 50,001 | SSC4D |  | 1.68453 | 1.33055 | 0.000643781 | 0.000128006 |
| Chromosome 19 | 100,001 | FZD9 | BM | 2.03333 | 0.752395 | 0.000493738 | 7.07E-05 |
| Chromosome 19 | 100,001 | FKBP6 |  | 2.03333 | 0.752395 | 0.000493738 | 7.07E-05 |
| Chromosome 19 | 100,001 | NSUN5 |  | 2.03333 | 0.752395 | 0.000493738 | 7.07E-05 |
| Chromosome 1A | 49,875,001 | SLC16A7 |  | 0.427532 | 0.776071 | 0.000295082 | 0.000445034 |
| Chromosome 1A | 64,025,001 | CHST11 | BM | 0.248406 | 1.24172 | 2.40E-05 | 1.50E-05 |
| Chromosome 1A | 64,025,001 | SLC41A2 | OR | 0.248406 | 1.24172 | 2.40E-05 | 1.50E-05 |
| Chromosome 2 | 43,650,001 | ENKUR |  | 1.69669 | -0.139678 | 0.000606771 | 0.000300354 |
| Chromosome 20 | 225,001 | RBM12 | OR | 0.651913 | 1.10586 | 3.60E-05 | 4.20E-05 |
| Chromosome 20 | 225,001 | ROMO1 |  | 0.651913 | 1.10586 | 3.60E-05 | 4.20E-05 |
| Chromosome 20 | 225,001 | RBM39 | BM | 0.651913 | 1.10586 | 3.60E-05 | 4.20E-05 |
| Chromosome 20 | 1,150,001 | AAR2 |  | 0.00586441 | 1.30676 | 0.000131008 | 0.000307699 |
| Chromosome 24 | 1,275,001 | TMPRSS4 |  | 0.840657 | 1.30499 | 0.00016901 | 0.000389374 |
| Chromosome 24 | 1,275,001 | SCN4B | OR | 0.840657 | 1.30499 | 0.00016901 | 0.000389374 |
| Chromosome 24 | 1,275,001 | SCN2B | OR | 0.840657 | 1.30499 | 0.00016901 | 0.000389374 |
| Chromosome 24 | 1,275,001 | MPZL2 |  | 0.840657 | 1.30499 | 0.00016901 | 0.000389374 |
| Chromosome 24 | 1,275,001 | ZW10 |  | 0.840657 | 1.30499 | 0.00016901 | 0.000389374 |
| Chromosome 24 | 1,675,001 | PTS |  | 1.1455 | 1.62116 | 0.000981938 | 0.000999162 |
| Chromosome 24 | 1,675,001 | IL18 |  | 1.1455 | 1.62116 | 0.000981938 | 0.000999162 |
| Chromosome 24 | 1,675,001 | DLAT |  | 1.1455 | 1.62116 | 0.000981938 | 0.000999162 |
| Chromosome 24 | 1,900,001 | LAYN |  | -0.264521 | 1.45689 | 8.00E-06 | 1.60E-05 |
| Chromosome 3 | 19,000,001 | HNRNPU | CR | -0.283306 | 0.26356 | 0.000258708 | 0.000283691 |
| Chromosome 3 | 19,000,001 | COX20 |  | -0.283306 | 0.26356 | 0.000258708 | 0.000283691 |
| Chromosome 3 | 19,000,001 | DESI2 |  | -0.283306 | 0.26356 | 0.000258708 | 0.000283691 |
| Chromosome 3 | 19,000,001 | ADSS |  | -0.283306 | 0.26356 | 0.000258708 | 0.000283691 |
| Chromosome 3 | 46,850,001 | TMEM14A | OR | 1.45689 | NA | 1.60E-05 | NA |
| Chromosome 4 | 17,375,001 | NAA15 |  | -0.0622515 | 0.534054 | 0.000320358 | 0.00037204 |
| Chromosome 4 | 17,375,001 | RAB33B |  | -0.0622515 | 0.534054 | 0.000320358 | 0.00037204 |
| Chromosome 4 | 17,500,001 | MAML3 |  | -1.72822 | 1.96045 | 7.70E-05 | 0.000505716 |
| Chromosome 4 | 58,900,001 | CYP2U1 |  | 0.663827 | 1.0731 | 0.000391092 | 0.000495728 |
| Chromosome 4 | 58,900,001 | HADH |  | 0.663827 | 1.0731 | 0.000391092 | 0.000495728 |
| Chromosome 4 | 58,900,001 | LEF1 | BM | 0.663827 | 1.0731 | 0.000391092 | 0.000495728 |
| Chromosome 4A | 7,625,001 | ARHGEF6 |  | -0.183715 | 2.01205 | 4.60E-05 | 0.000145668 |
| Chromosome 5 | 17,225,001 | CPT1A | CR | 0.744762 | 2.0933 | 0.000560884 | 0.000712408 |
| Chromosome 5 | 17,225,001 | EHF |  | 0.744762 | 2.0933 | 0.000560884 | 0.000712408 |
| Chromosome 5 | 17,375,001 | APIP |  | 1.05642 | 0.945118 | 0.000954348 | 0.000818808 |
| Chromosome 5 | 17,375,001 | PDHX |  | 1.05642 | 0.945118 | 0.000954348 | 0.000818808 |
| Chromosome 7 | 16,425,001 | GLI2 |  | 0.255634 | 2.19849 | 0.000173345 | 0.000422711 |
| Unmapped | 4,225,001 | ARHGAP5 | OR | 0.0495758 | 0.381009 | 2.93E-05 | 1.10E-05 |
| Z Chromosome | 15,025,001 | SLC28A3 | OR | -0.0220229 | 1.88439 | 3.80E-05 | 0.000427679 |
| Z Chromosome | 15,050,001 | RMI1 |  | -1.08981 | 1.31977 | 0.000103009 | 0.000473027 |
| Z Chromosome | 20,750,001 | PPIP5K2 | OR | 0.736107 | -0.51131 | 7.80E-05 | 6.00E-05 |
| Z Chromosome | 20,750,001 | GIN1 |  | 0.736107 | -0.51131 | 7.80E-05 | 6.00E-05 |
| Z Chromosome | 20,800,001 | PAM |  | 2.01245 | -0.558812 | 0.00018737 | 0.000134003 |
| Z Chromosome | 29,825,001 | TMEM161B | OR | 0.783789 | -1.40348 | 0.000116344 | 9.23E-05 |
| Z Chromosome | 30,050,001 | CCNH |  | 2.0443 | 0.462648 | 4.30E-05 | 6.20E-05 |
| Z Chromosome | 30,050,001 | RASA1 | OR | 2.0443 | 0.462648 | 4.30E-05 | 6.20E-05 |
| Z Chromosome | 32,400,001 | RFX3 |  | 1.38919 | 0.224272 | 0.000230055 | 0.000301681 |
| Z Chromosome | 35,800,001 | AGGF1 |  | 1.70719 | 0.662173 | 0.000461762 | 0.000302018 |
| Z Chromosome | 35,825,001 | PDE8B |  | 2.31438 | 1.26727 | 0.000111335 | 0.000289678 |
| Z Chromosome | 35,850,001 | WDR41 |  | 2.31785 | 0.970675 | 0.000172356 | 0.000413369 |
| Z Chromosome | 36,000,001 | TBCA |  | -0.12555 | 1.52208 | 0.00012936 | 0.000513039 |
| Z Chromosome | 36,275,001 | AP3B1 |  | 1.42302 | 1.76302 | 5.57E-05 | 0.000259674 |
| Z Chromosome | 36,275,001 | SCAMP1 |  | 1.42302 | 1.76302 | 5.57E-05 | 0.000259674 |
| Z Chromosome | 36,875,001 | PAPD4 |  | 0.81873 | 1.36964 | 0.000453532 | 0.000833747 |
| Z Chromosome | 44,375,001 | HSPB3 |  | 1.24321 | 1.90448 | 9.30E-05 | 0.000501057 |
| Z Chromosome | 65,125,001 | BNC2 |  | 0.458782 | 0.197268 | 4.07E-05 | 0.000266682 |

**Extended Data Table 7:** Candidate genes identified through whole genome comparisons of freshwater and salt water populations of Song Sparrows. Genes are linked to potential adaptive functions, including osmoregulatory (OR), kidney development (KD), circadian rhythm (CR), bill morphology (BM), melanin pathways (MP), or response to heat stress (HS). Window-based Tajima’s D and pi estimates are presented for both coastal and interior populations. The strongest candidates (elevated *F*_ST_ and negative Tajima’s D in one population) are highlighted in grey, genes that are shared with at least one other species pair are highlighted in blue.

| **Chromosome** | **Position Start** | **Gene ID** | **Putative Adaptive Function** | **Tajima's D Coastal** | **Tajima's D Interior** | **Pi Coastal** | **Pi Interior** |
| --- | --- | --- | --- | --- | --- | --- | --- |
| Chromosome 1 | 15,350,001 | CDKL5 |  | -0.59155 | 1.35442 | 7.58E-06 | 1.26E-05 |
| Chromosome 1 | 16,900,001 | ASB11 |  | 1.52343 | -1.09741 | 1.68E-05 | 3.58E-06 |
| Chromosome 1 | 34,325,001 | BRCA2 |  | 1.56503 | NA | 1.35E-05 | NA |
| Chromosome 1 | 70,500,001 | TMEM131 | OR | 1.19062 | NA | 1.52E-05 | NA |
| Chromosome 1 | 70,500,001 | INPP4A | OR | 1.19062 | NA | 1.52E-05 | NA |
| Chromosome 1 | 75,500,001 | GAS6 |  | 1.19062 | NA | 1.52E-05 | NA |
| Chromosome 1 | 75,500,001 | TMEM255B | OR | 1.19062 | NA | 1.52E-05 | NA |
| Chromosome 1 | 103,725,001 | NME7 |  | -0.473404 | 0.899417 | 6.74E-06 | 1.37E-05 |
| Chromosome 1 | 103,725,001 | ATP1B1 | OR | -0.473404 | 0.899417 | 6.74E-06 | 1.37E-05 |
| Chromosome 1 | 114,375,001 | TMPRSS7 | OR | -1.1407 | 1.89655 | 1.16E-05 | 2.61E-05 |
| Chromosome 1 | 114,375,001 | ABHD10 |  | -1.1407 | 1.89655 | 1.16E-05 | 2.61E-05 |
| Chromosome 10 | 15,575,001 | DIS3L |  | 1.39862 | -0.59155 | 1.62E-05 | 7.58E-06 |
| Chromosome 11 | 3,375,001 | FOXF1 |  | 1.89106 | NA | 2.95E-05 | 0.000773419 |
| Chromosome 12 | 11,075,001 | SLC41A3 | OR | 0.248757 | 1.13587 | 8.21E-06 | 3.35E-05 |
| Chromosome 12 | 15,275,001 | PCBP4 |  | 0.722614 | 0.351953 | 1.01E-05 | 1.35E-05 |
| Chromosome 12 | 15,275,001 | ABHD14B |  | 0.722614 | 0.351953 | 1.01E-05 | 1.35E-05 |
| Chromosome 12 | 15,275,001 | SEMA3B |  | 0.722614 | 0.351953 | 1.01E-05 | 1.35E-05 |
| Chromosome 12 | 15,275,001 | SEMA3F |  | 0.722614 | 0.351953 | 1.01E-05 | 1.35E-05 |
| Chromosome 12 | 15,275,001 | SEMA3G |  | 0.722614 | 0.351953 | 1.01E-05 | 1.35E-05 |
| Chromosome 12 | 15,275,001 | BAP1 |  | 0.722614 | 0.351953 | 1.01E-05 | 1.35E-05 |
| Chromosome 12 | 15,275,001 | DNAH1 |  | 0.722614 | 0.351953 | 1.01E-05 | 1.35E-05 |
| Chromosome 12 | 18,175,001 | IPPK | OR | 1.09117 | -0.0861022 | 1.16E-05 | 1.07E-05 |
| Chromosome 12 | 18,175,001 | OGN | BM | 1.09117 | -0.0861022 | 1.16E-05 | 1.07E-05 |
| Chromosome 12 | 18,175,001 | CENPP |  | 1.09117 | -0.0861022 | 1.16E-05 | 1.07E-05 |
| Chromosome 13 | 2,250,001 | ABLIM3 | OR | -0.404661 | 0.570764 | 2.08E-05 | 4.11E-05 |
| Chromosome 14 | 125,001 | ROGDI |  | 1.52343 | NA | 1.68E-05 | NA |
| Chromosome 14 | 125,001 | GLYR1 | OR | 1.52343 | NA | 1.68E-05 | NA |
| Chromosome 14 | 125,001 | MRPS34 |  | 1.52343 | NA | 1.68E-05 | NA |
| Chromosome 14 | 125,001 | MAPK8IP3 | OR | 1.52343 | NA | 1.68E-05 | NA |
| Chromosome 1A | 5,450,001 | SFMBT2 |  | 0.899417 | 0.525011 | 1.37E-05 | 1.18E-05 |
| Chromosome 1A | 23,725,001 | CD9 | OR | 0.828692 | -0.473404 | 4.74E-05 | 6.74E-06 |
| Chromosome 1A | 23,725,001 | FAR2 |  | 0.828692 | -0.473404 | 4.74E-05 | 6.74E-06 |
| Chromosome 1A | 44,025,001 | FOXP2 |  | -0.31822 | 1.63934 | 1.49E-05 | 3.83E-05 |
| Chromosome 1A | 51,300,001 | SRGAP | OR | NA | 1.52852 | NA | 3.07E-05 |
| Chromosome 1A | 51,300,001 | RPL18A |  | NA | 1.52852 | NA | 3.07E-05 |
| Chromosome 1A | 51,300,001 | XPOT |  | NA | 1.52852 | NA | 3.07E-05 |
| Chromosome 2 | 1,325,001 | SETD2 |  | NA | 1.19062 | NA | 1.52E-05 |
| Chromosome 2 | 28,075,001 | NPVF |  | 1.35442 | NA | 1.26E-05 | NA |
| Chromosome 2 | 43,500,001 | KIAA1217 |  | NA | 1.53133 | NA | 2.08E-05 |
| Chromosome 2 | 74,125,001 | ATP9B |  | 0.722614 | -0.0861022 | 1.01E-05 | 1.07E-05 |
| Chromosome 2 | 105,200,001 | METTL4 |  | NA | -0.0861022 | NA | NA |
| Chromosome 3 | 12,250,001 | PLCB4 | OR | -1.01486 | 1.43024 | 3.16E-06 | 2.02E-05 |
| Chromosome 3 | 34,050,001 | HHAT |  | 0.899417 | -0.473404 | 1.37E-05 | 6.74E-06 |
| Chromosome 3 | 39,300,001 | RPS6KA2 |  | 1.51238 | -0.59155 | 1.33E-05 | 7.58E-06 |
| Chromosome 4 | 100,001 | USO1 |  | 1.22093 | NA | 2.44E-05 | NA |
| Chromosome 4 | 100,001 | AREG |  | 1.22093 | NA | 2.44E-05 | NA |
| Chromosome 4 | 100,001 | EREG |  | 1.22093 | NA | 2.44E-05 | NA |
| Chromosome 4 | 100,001 | EPGN |  | 1.22093 | NA | 2.44E-05 | NA |
| Chromosome 4 | 23,600,001 | SMIM20 |  | NA | 1.26176 | NA | 1.92E-05 |
| Chromosome 4 | 23,600,001 | SEL1L3 |  | NA | 1.26176 | NA | 1.92E-05 |
| Chromosome 4 | 26,200,001 | LCORL |  | NA | 1.26176 | NA | 4.51E-05 |
| Chromosome 4 | 26,200,001 | NCAPG |  | NA | 1.26176 | NA | 4.51E-05 |
| Chromosome 4 | 27,100,001 | CC2D2A |  | 1.5586 | 0.384848 | 6.32E-05 | 2.13E-05 |
| Chromosome 4 | 40,525,001 | KIAA1456 |  | 1.75668 | NA | 4.19E-05 | NA |
| Chromosome 4 | 40,525,001 | DLC1 | OR | 1.75668 | NA | 4.19E-05 | NA |
| Chromosome 4 | 54,475,001 | GUCY1A3 | OR | -1.09741 | 1.52343 | 3.58E-06 | 1.68E-05 |
| Chromosome 4A | 13,225,001 | COL4A5 |  | 0.903239 | 0.413494 | 4.53E-05 | 2.08E-05 |
| Chromosome 5 | 53,200,001 | GNG2 |  | 1.09117 | 0.722614 | 1.16E-05 | 1.58E-05 |
| Chromosome 6 | 20,625,001 | MYOF | OR, HS | 1.45284 | 0.706469 | 0.000371885 | 0.000378157 |
| Chromosome 6 | 20,625,001 | CEP55 |  | 1.45284 | 0.706469 | 0.000371885 | 0.000378157 |
| Chromosome 6 | 20,875,001 | PLCE1 | OR | -0.473404 | -0.473404 | 6.74E-06 | 0.000332431 |
| Chromosome 6 | 21,150,001 | HECTD2 |  | 1.35716 | -0.40873 | 0.000114955 | 0.000769576 |
| Chromosome 9 | 19,800,001 | TBL1XR1 |  | -1.16439 | 1.56503 | 4.00E-06 | 1.35E-05 |
| Z chromosome | 31,275,001 | GLDC |  | 1.56503 | NA | 1.35E-05 | NA |
| Z chromosome | 33,775,001 | MAP1B |  | 1.09117 | -0.0861022 | 1.16E-05 | 1.07E-05 |
| Z chromosome | 33,775,001 | MRPS27 |  | 1.09117 | -0.0861022 | 1.16E-05 | 1.07E-05 |
| Z chromosome | 33,775,001 | PTCD2 | KD | 1.09117 | -0.0861022 | 1.16E-05 | 1.07E-05 |
| Z chromosome | 34,000,001 | FCHO2 |  | 0.669155 | 1.24552 | 0.000435546 | 0.000457934 |
| Z chromosome | 35,125,001 | HMGCR |  | NA | 1.19062 | NA | 1.52E-05 |
| Z chromosome | 35,125,001 | COL4A3BP |  | NA | 1.19062 | NA | 1.52E-05 |
| Z chromosome | 35,125,001 | POLK |  | NA | 1.19062 | NA | 1.52E-05 |
| Z chromosome | 37,475,001 | PIK3R1 |  | 0.248757 | 0.938857 | 8.21E-06 | 3.16E-05 |
| Z chromosome | 42,700,001 | NDUFS4 |  | 1.39862 | 0.0674041 | 1.62E-05 | 9.47E-06 |
| Z chromosome | 44,925,001 | NNT |  | 1.45909 | 0.172703 | 2.69E-05 | 2.42E-05 |
| Z chromosome | 48,625,001 | ABCA1 |  | NA | 1.26176 | NA | 1.92E-05 |
| Z chromosome | 58,250,001 | C9 |  | 1.09117 | 1.54631 | 1.16E-05 | 1.58E-05 |
| Z chromosome | 58,250,001 | DAB2 | OR | 1.09117 | 1.54631 | 1.16E-05 | 1.58E-05 |

**Extended Data Table 8:** Candidate genes identified through whole genome comparisons of freshwater and salt water populations of Swamp Sparrows. Genes are linked to potential adaptive functions, including osmoregulatory (OR), kidney development (KD), circadian rhythm (CR), bill morphology (BM), melanin pathways (MP), or response to heat stress (HS). Window-based Tajima’s D and pi estimates are presented for both coastal and interior populations. The strongest candidates (elevated *F*_ST_ and negative Tajima’s D in one population) are highlighted in grey, genes that are shared with at least one other species pair are highlighted in blue.

| **Chromosome** | **Position Start** | **Gene ID** | **Putative Adaptive Function** | **Tajima's D Coastal** | **Tajima's D Interior** | **Pi Coastal** | **Pi Interior** |
| --- | --- | --- | --- | --- | --- | --- | --- |
| Chromosome 10 | 19,200,001 | BNIP2 |  | 0.275278 | 2.63748 | 0.000513497 | 0.00215067 |
| Chromosome 10 | 19,200,001 | GTF2A2 |  | 0.275278 | 2.63748 | 0.000513497 | 0.00215067 |
| Chromosome 10 | 20,775,001 | PDIA3 |  | 0.84746 | 1.05373 | 0.00222361 | 0.00234789 |
| Chromosome 10 | 20,775,001 | PPIP5K1 | OR | 0.84746 | 1.05373 | 0.00222361 | 0.00234789 |
| Chromosome 10 | 20,825,001 | TUBGCP4 |  | -0.44469 | 1.81467 | 0.000262532 | 0.000504377 |
| Chromosome 14 | 150,001 | GLYR1 |  | -0.286989 | 1.07612 | 0.000211793 | 2.96E-05 |
| Chromosome 14 | 150,001 | MRPS34 |  | -0.286989 | 1.07612 | 0.000211793 | 2.96E-05 |
| Chromosome 14 | 150,001 | MAPK8IP3 | OR | -0.286989 | 1.07612 | 0.000211793 | 2.96E-05 |
| Chromosome 1A | 21,250,001 | CYB5R3 | OR | 2.76638 | 3.03879 | 0.00288893 | 0.00460789 |
| Chromosome 1A | 21,250,001 | LRMP |  | 2.76638 | 3.03879 | 0.00288893 | 0.00460789 |
| Chromosome 1A | 21,850,001 | TTLL1 |  | 0.673161 | 1.21886 | 0.0020478 | 0.00227341 |
| Chromosome 1A | 21,850,001 | MCAT |  | 0.673161 | 1.21886 | 0.0020478 | 0.00227341 |
| Chromosome 1A | 21,850,001 | TSPO | OR | 0.673161 | 1.21886 | 0.0020478 | 0.00227341 |
| Chromosome 1A | 21,850,001 | TTLL12 |  | 0.673161 | 1.21886 | 0.0020478 | 0.00227341 |
| Chromosome 1A | 21,850,001 | SCUBE1 |  | 0.673161 | 1.21886 | 0.0020478 | 0.00227341 |
| Chromosome 1A | 64,125,001 | SLC41A2 | OR | 3.05696 | 1.00345 | 0.000375588 | 0.000296381 |
| Chromosome 1A | 64,125,001 | C12orf45 |  | 3.05696 | 1.00345 | 0.000375588 | 0.000296381 |
| Chromosome 1A | 64,125,001 | WASHC4 |  | 3.05696 | 1.00345 | 0.000375588 | 0.000296381 |
| Chromosome 1A | 50,001 | SMO |  | 1.11564 | 1.19045 | 0.003502 | 0.00325817 |
| Chromosome 2 | 45,350,001 | VPS41 | OR | 1.26649 | 1.29855 | 0.0037962 | 0.00467384 |
| Chromosome 2 | 74,650,001 | ADNP2 |  | 2.72516 | 0.778643 | 0.000500651 | 0.000333928 |
| Chromosome 2 | 74,650,001 | RECK |  | 2.72516 | 0.778643 | 0.000500651 | 0.000333928 |
| Chromosome 2 | 74,725,001 | TGFBR1 | OR | 1.58308 | -0.95386 | 0.000163789 | 6.72E-05 |
| Chromosome 2 | 74,925,001 | TRIP13 |  | 1.85824 | -1.51469 | 4.04E-05 | 6.67E-06 |
| Chromosome 2 | 75,150,001 | SLC9A3 | OR | 2.81515 | -1.51469 | 0.000343797 | 6.67E-06 |
| Chromosome 2 | 75,175,001 | LRRC14B |  | 2.32511 | -1.27479 | 0.000258105 | 2.13E-05 |
| Chromosome 2 | 76,250,001 | PPP1R17 |  | 2.19012 | -1.17279 | 0.000312852 | 3.13E-05 |
| Chromosome 2 | 158,625,001 | FAM83H | BM | 1.65569 | 0.30588 | 0.00155734 | 0.00102893 |
| Chromosome 20 | 100,001 | RBM12 | OR | 2.30027 | 1.39025 | 0.000224218 | 0.00016986 |
| Chromosome 20 | 250,001 | ROMO1 |  | 0.785559 | 1.85153 | 0.00024885 | 0.000304074 |
| Chromosome 20 | 250,001 | RBM39 | BM | 0.785559 | 1.85153 | 0.00024885 | 0.000304074 |
| Chromosome 20 | 350,001 | PHF20 |  | -0.076963 | 1.31307 | 0.000106317 | 0.00017377 |
| Chromosome 20 | 425,001 | NDRG3 |  | 1.47316 | 0.119221 | 0.000530117 | 0.000407702 |
| Chromosome 20 | 14,900,001 | STAU1 |  | 0.74657 | 1.22457 | 0.000133264 | 0.000157537 |
| Chromosome 20 | 14,900,001 | STAU2 |  | 0.74657 | 1.22457 | 0.000133264 | 0.000157537 |
| Chromosome 20 | 14,900,001 | CSE1L |  | 0.74657 | 1.22457 | 0.000133264 | 0.000157537 |
| Chromosome 20 | 14,900,001 | ARFGEF2 |  | 0.74657 | 1.22457 | 0.000133264 | 0.000157537 |
| Chromosome 20 | 14,900,001 | ARFGEF1 |  | 0.74657 | 1.22457 | 0.000133264 | 0.000157537 |
| Chromosome 20 | 15,025,001 | PREX1 |  | -0.20733 | 1.63499 | 7.07E-05 | 0.000164932 |
| Chromosome 20 | 15,200,001 | SULF2 | BM | 0.638649 | 1.47029 | 0.000159368 | 0.000181892 |
| Chromosome 20 | 15,200,001 | NCOA3 |  | 0.638649 | 1.47029 | 0.000159368 | 0.000181892 |
| Chromosome 23 | 25,001 | YTHDF2 |  | -0.277059 | 1.7559 | 0.000209903 | 0.00039639 |
| Chromosome 23 | 25,001 | GMEB1 |  | -0.277059 | 1.7559 | 0.000209903 | 0.00039639 |
| Chromosome 23 | 25,001 | TAF12 |  | -0.277059 | 1.7559 | 0.000209903 | 0.00039639 |
| Chromosome 23 | 25,001 | TRNAU1AP |  | -0.277059 | 1.7559 | 0.000209903 | 0.00039639 |
| Chromosome 24 | 75,001 | DPAGT1 |  | -0.0780472 | 1.5593 | 7.62E-05 | 0.00172206 |
| Chromosome 24 | 75,001 | HMBS |  | -0.0780472 | 1.5593 | 7.62E-05 | 0.00172206 |
| Chromosome 24 | 75,001 | VPS11 | OR | -0.0780472 | 1.5593 | 7.62E-05 | 0.00172206 |
| Chromosome 24 | 75,001 | DDX6 |  | -0.0780472 | 1.5593 | 7.62E-05 | 0.00172206 |
| Chromosome 24 | 75,001 | CXCR5 |  | -0.0780472 | 1.5593 | 7.62E-05 | 0.00172206 |
| Chromosome 24 | 175,001 | CCDC84 |  | 1.51653 | 0.576794 | 0.000512675 | 0.000306391 |
| Chromosome 24 | 175,001 | RPS25 |  | 1.51653 | 0.576794 | 0.000512675 | 0.000306391 |
| Chromosome 24 | 175,001 | TRAPPC4 |  | 1.51653 | 0.576794 | 0.000512675 | 0.000306391 |
| Chromosome 24 | 175,001 | SLC37A4 |  | 1.51653 | 0.576794 | 0.000512675 | 0.000306391 |
| Chromosome 24 | 175,001 | HYOU1 |  | 1.51653 | 0.576794 | 0.000512675 | 0.000306391 |
| Chromosome 24 | 175,001 | PHLDB1 |  | 1.51653 | 0.576794 | 0.000512675 | 0.000306391 |
| Chromosome 24 | 175,001 | IFT46 |  | 1.51653 | 0.576794 | 0.000512675 | 0.000306391 |
| Chromosome 24 | 175,001 | KMT2A | CR | 1.51653 | 0.576794 | 0.000512675 | 0.000306391 |
| Chromosome 28 | 550,001 | SLC1A6 | OR | 1.40689 | 1.87562 | 0.00447402 | 0.00465819 |
| Chromosome 28 | 550,001 | RANBP3 |  | 1.40689 | 1.87562 | 0.00447402 | 0.00465819 |
| Chromosome 28 | 550,001 | KLHL33 |  | 1.40689 | 1.87562 | 0.00447402 | 0.00465819 |
| Chromosome 3 | 3,350,001 | C1D |  | 0.670507 | -0.599058 | 4.23E-05 | 5.80E-06 |
| Chromosome 3 | 3,350,001 | PNO1 |  | 0.670507 | -0.599058 | 4.23E-05 | 5.80E-06 |
| Chromosome 3 | 3,350,001 | CNRIP1 | OR | 0.670507 | -0.599058 | 4.23E-05 | 5.80E-06 |
| Chromosome 3 | 3,650,001 | BFSP1 |  | -0.016499 | 0.344844 | 0.000405477 | 2.48E-05 |
| Chromosome 3 | 3,650,001 | PCSK2 |  | -0.016499 | 0.344844 | 0.000405477 | 2.48E-05 |
| Chromosome 3 | 28,825,001 | SUSD4 |  | -0.768842 | 0.268176 | 1.43E-05 | 1.04E-05 |
| Chromosome 3 | 29,050,001 | DUSP10 | OR | 0.421151 | 0.138693 | 3.92E-05 | 1.16E-05 |
| Chromosome 3 | 29,150,001 | TAF1A |  | 1.19062 | NA | 1.52E-05 | NA |
| Chromosome 4 | 40,425,001 | AASDH |  | 1.49546 | -1.17434 | 0.000203791 | 1.49E-05 |
| Chromosome 4 | 40,425,001 | LONRF1 |  | 1.49546 | -1.17434 | 0.000203791 | 1.49E-05 |
| Chromosome 4 | 40,575,001 | KIAA1456 |  | 0.842162 | 0.133221 | 8.23E-05 | 6.65E-05 |
| Chromosome 4 | 40,575,001 | DLC1 | OR | 0.842162 | 0.133221 | 8.23E-05 | 6.65E-05 |
| Chromosome 5 | 3,875,001 | LGR4 | OR | 0.895077 | -1.11398 | 3.12E-05 | 3.04E-06 |
| Chromosome 5 | 3,875,001 | LIN7C |  | 0.895077 | -1.11398 | 3.12E-05 | 3.04E-06 |
| Chromosome 5 | 4,300,001 | METTL15 |  | 0.285403 | 1.62071 | 0.000459176 | 0.000641934 |
| Chromosome 5 | 10,800,001 | TNNI2 |  | 1.04664 | 1.93918 | 0.00119329 | 0.00215475 |
| Chromosome 5 | 10,800,001 | SYT8 |  | 1.04664 | 1.93918 | 0.00119329 | 0.00215475 |
| Chromosome 5 | 10,800,001 | IFITM10 |  | 1.04664 | 1.93918 | 0.00119329 | 0.00215475 |
| Chromosome 5 | 49,000,001 | SPRED1 | OR | -2.25067 | 2.60647 | 7.12E-05 | 0.000319863 |
| Chromosome 5 | 49,025,001 | FAM98B |  | -1.93636 | 2.61398 | 5.03E-05 | 0.000258552 |
| Chromosome 5 | 49,050,001 | RASGRP1 | OR | -1.72331 | 3.02838 | 1.20E-05 | 0.000220291 |
| Chromosome 5 | 49,525,001 | RYR3 |  | 1.13587 | NA | 3.35E-05 | NA |
| Chromosome 5 | 49,625,001 | AVEN |  | NA | 0.480279 | NA | 1.38E-05 |
| Chromosome 5 | 49,675,001 | CHRM4 | OR | 1.02588 | NA | 1.77E-05 | NA |
| Chromosome 5 | 49,675,001 | EMC7 |  | 1.02588 | NA | 1.77E-05 | NA |
| Chromosome 6 | 21,750,001 | ACTR1A |  | 2.82235 | 0.463222 | 0.00581048 | 0.00361802 |
| Chromosome 6 | 21,750,001 | LIPA |  | 2.82235 | 0.463222 | 0.00581048 | 0.00361802 |
| Chromosome 6 | 21,750,001 | TMEM72 | OR | 2.82235 | 0.463222 | 0.00581048 | 0.00361802 |
| Chromosome 6 | 22,275,001 | ERCC6 |  | -0.198544 | 1.08147 | 0.000501103 | 0.000725703 |
| Chromosome 6 | 22,275,001 | DRGX | HS | -0.198544 | 1.08147 | 0.000501103 | 0.000725703 |
| Chromosome 6 | 23,300,001 | MARCH8 |  | 0.910757 | 1.26467 | 0.00239193 | 0.00239658 |
| Chromosome 6 | 23,325,001 | BLOC1S2 | MP | 0.556054 | 1.96759 | 0.00290621 | 0.00346002 |
| Chromosome 6 | 23,325,001 | PDK2L1 | OR | 0.556054 | 1.96759 | 0.00290621 | 0.00346002 |
| Chromosome 7 | 9,475,001 | MARCH7 |  | 0.940846 | 2.41743 | 0.000333268 | 0.000416111 |
| Chromosome 8 | 21,750,001 | ATP6V1G3 | OR | -0.551579 | 2.11669 | 0.00393435 | 0.00644239 |
| Chromosome 8 | 25,525,001 | BRINP2 |  | -0.111115 | 2.38072 | 0.00289102 | 0.00469179 |
| Chromosome 9 | 25,850,001 | TM4SF4 |  | 1.8128 | 1.69452 | 0.00037264 | 0.00054205 |
| Chromosome 9 | 25,850,001 | HPS3 | MP | 1.8128 | 1.69452 | 0.00037264 | 0.00054205 |
| Chromosome 9 | 25,850,001 | GYG1 |  | 1.8128 | 1.69452 | 0.00037264 | 0.00054205 |
| unmapped | 4,200,001 | ARGHAP5 |  | 1.74445 | -1.40825 | 6.53E-05 | 1.19E-05 |
| unmapped | 12,050,001 | DNM1 |  | 2.40719 | 0.981273 | 7.90E-05 | 3.75E-05 |
| Z Chromosome | 9,575,001 | ELAVL2 |  | 0.871318 | 0.660888 | 0.0018488 | 0.00154652 |
| Z Chromosome | 10,675,001 | UGCG |  | 1.39987 | 1.36579 | 0.00141104 | 0.00149989 |
| Z Chromosome | 15,200,001 | IDNK |  | 0.261709 | 1.44472 | 0.00138122 | 0.00166443 |
| Z Chromosome | 22,450,001 | STARD4 |  | 0.775981 | 0.887968 | 0.00132602 | 0.00125288 |
| Z Chromosome | 22,575,001 | EPB41L4A | OR | 0.467991 | 1.64965 | 0.000896442 | 0.00113503 |
| Z Chromosome | 46,500,001 | PCGF3 |  | 0.305494 | 1.5975 | 0.000750111 | 0.00112557 |
| Z Chromosome | 46,500,001 | MFSD7 |  | 0.305494 | 1.5975 | 0.000750111 | 0.00112557 |
| Z Chromosome | 47,575,001 | HOOK3 |  | 1.18116 | 0.792014 | 0.00142686 | 0.00122619 |
| Z Chromosome | 47,575,001 | KCMF1 |  | 1.18116 | 0.792014 | 0.00142686 | 0.00122619 |
| Z Chromosome | 48,425,001 | LPL |  | 0.999428 | 1.19582 | 0.000913377 | 0.00114406 |
| Z Chromosome | 50,075,001 | ROR2 | BM | 0.939128 | 1.01788 | 0.00256579 | 0.00253669 |
| Z Chromosome | 50,075,001 | NFIL3 | CR | 0.939128 | 1.01788 | 0.00256579 | 0.00253669 |
| Z Chromosome | 56,075,001 | TARS |  | 0.752934 | 0.728598 | 0.00117584 | 0.00112007 |
| Z Chromosome | 58,200,001 | RICTOR |  | 0.730671 | 0.969248 | 0.00192164 | 0.00203553 |
| Z Chromosome | 58,275,001 | C9 |  | 0.87446 | 2.07763 | 0.000626957 | 0.000733087 |
| Z Chromosome | 58,275,001 | DAB2 | OR | 0.87446 | 2.07763 | 0.000626957 | 0.000733087 |
| Z Chromosome | 59,150,001 | FBXO4 |  | 1.10328 | 0.623278 | 0.000382758 | 0.000346397 |
| Z Chromosome | 75,875,001 | MELK |  | 3.03505 | 0.503147 | 0.000794768 | 0.000431042 |
